# Supplementary material for: Evaluation of the efficacy and safety of conventional and interlaminar full-endoscopic decompressive laminectomy to treat lumbar spinal stenosis (ENDO-F trial): Protocol for a prospective, randomized, multicenter trial
Source: PLoS One. 2023 Apr 5;18(4):e0283924. doi: 10.1371/journal.pone.0283924 (PMC10075401; doi:10.1371/journal.pone.0283924)
Supplement: S2 File — (DOCX) [file pone.0283924.s002.docx]

STUDY PROTOCOL

(For a clinical study involving other types of medical interventions (surgery, radiation therapy, etc.))

1. Study Title

국문: 요추부 협착증에서 lamina간 경유 경피적 단일공 내시경하 후방감압술과 conventional 후방 감압술의 임상적 및 방사선학적 efficacy 및 안전성 평가 (ENDO-F Trial): 전향적, 무작위 배정, 평가자 눈가림, 다기관 연구

영문: Evaluation of the efficacy and safety of conventional and interlaminar full-endoscopic decompressive laminectomy to treat lumbar spinal stenosis (ENDO-F Trial): A prospective, randomized, Assessor blind, multicenter trial

1. Name and Address of Study Institutions

| Name of Institution | Address |
| --- | --- |
| Seoul St Mary’s Hospital,  The Catholic University of Korea | Department of Neurosurgery, Seoul St Mary’s Hospital of The Catholic University of Korea, 222, Banpo-daero, Banpo-dong, Seocho-gu, Seoul |
| Chungdam Wooridul Spine Hospital | 47-4, Cheongdam-dong, Gangnam-gu, Seoul |
| Wiltse Memorial Hospital (Anyang) | 560, Gyeongsu-daero, Dongan-gu, Anyang-si (Hogye-dong) |

1. List of Investigators

|  | Seoul St Mary’s Hospital | Chungdam Wooridul Spine Hospital | Wiltse Memorial Hospital (Anyang) |
| --- | --- | --- | --- |
| Principal Investigator | Prof. Jin-Sung Kim **(Coordinating Investigator)** | Junseok Bae, President of Hospital | Dong Chan Lee, President of Hospital |
| Co-investigators | Woojung Lim, Clinical Instructor  Junghoon Kim, Clinical Instructor | Sang-Ha Shin, Director  Sang Soo Eun, Director  Han Joong Keum, Director  Young Soo Choi, Director | Tae Hyun Kim, Director  Seung Ho Shin, Director  Hyun Jin Hong, Director  Ji Yeon Kim, Direcctor |
| Subinvestigators | Eun Kim , Researcher  Cho-Rong Lee , Researcher | Yun-Ju Lee , Researcher | Do-Yeon Kim , Researcher |

1. Name and Address of Study Sponsor (Sponsor Institution)

Jin-Sung Kim, Department of Neurosurgery, Seoul St Mary’s Hospital, The Catholic University of Korea

1. Study Objectives

This study is a randomized clinical trial (RCT) that aims to verify the equivalence of clinical outcomes of interlaminar full-endoscopic laminectomy to those of a conventional surgical technique (open decompressive laminectomy) for patients with lumbar spinal stenosis. In addition, the prospective clinical data of interlaminar full-endoscopic laminectomy will be analyzed to establish the basis for clinical practice guidelines and present a guideline for selection of treatment methods in clinical practice. To this end, the clinical efficacy and safety of posterior decompressive laminectomy using the full-endoscopic uniportal technique in patients with lumbar spinal stenosis of three study institutions were compared with those of the conventional surgical method.

1. Study Background

According to the Health Insurance Review and Assessment Service (HIRA), the number of patients with spinal diseases, the target population of this study, was reported to be around 3.63 million in 2018 alone, showing an increase of 450,000 patients over the past five years. In particular, the number of patients with lumbar spinal stenosis (LSS) increased by approximately 32.4% in 5 years. According to the 2018 Statistical Yearbook of Surgery by the National Health Insurance, there were 7,218 cases of endoscopic spine surgery, an increase of 41.3% from 5,108 cases in the previous year, and the treatment cost also increased by 45.9%. Compared to the increase of 2.4% in the number of conventional spine surgeries from 165,573 cases to 169,706 cases for the same period, a significant increase is confirmed, indicating that the cases of endoscopic spine surgery show a trend of remarkable growth. In addition, the prevalence of spinal diseases is expected to gradually escalate with the increase in the senior population, and the burden of increased medical expenses for the treatment is also projected.

Posterior decompression in lumbar spinal stenosis and posterior lumbar discectomy in lumbar disc herniation are the most conventional methods used to resolve the patient's symptoms. The conventional methods have problems in terms of much bleeding, postoperative pain, instability and a decrease of muscles around the spine, and therefore, minimally invasive surgery is performed to preserve anatomical structures. Unilateral laminectomy bilateral decompression (ULBD) is the representative most commonly used minimally invasive technique, and other methods such as spinous process osteotomy and endoscopy are also used. It has been reported that minimally invasive surgery has a number of advantages compared to the conventional surgical technique, and the clinical outcomes are not different from those of the conventional open laminectomy.

Recently, surgical techniques of spinal decompression and discectomy using endoscopy have been developed and applied in clinical practice. The endoscopic spine surgery is classified into uniportal endoscopic technique and biportal endoscopic technique depending on the number of ports for insertion of instruments. Since the surgical site is accessed through a skin incision of less than 1 cm, damage to the normal structure can be minimized, resulting in fewer postoperative complications such as pain and epidural adhesion.

However, although interlaminar full-endoscopic laminectomy has been reported with good clinical outcomes in retrospective studies, the clinical outcomes have not been confirmed in a multicenter, prospective, randomized clinical trial. In addition, while the conventional open laminectomy and microscopic surgery currently performed on patients with lumbar spinal stenosis are covered by national health insurance and are appropriately applied for patients in need of surgical treatments, endoscopic spine surgery are not fully accepted for health insurance coverage, and thus interlaminar full-endoscopic laminectomy has been performed only for some of the patients with lumbar disc herniation. Most of the interlaminar full-endoscopic laminectomy studies reported in Korea are retrospective studies, and there has been no level 1 study to establish the efficacy and safety of the endoscopic surgery compared to the conventional open laminectomy . Therefore, in this study, the efficacy and safety of interlaminar full-endoscopic laminectomy were compared with those of conventional open laminectomy with established clinical outcomes through a multicenter, prospective randomized trial.

1. Study Subjects

Patients with ≥ grade B lumbar central canal stenosis who agree to undergo one to two segment posterior spinal decompression surgery

Those who are willing to participate and fully comply with the 1-year follow-up protocol

1. Inclusion and Exclusion Criteria, Target Sample Size and Rationale for the Sample Size Determination

Inclusion and Exclusion Criteria

**# Patients with lumbar spinal stenosis**

1. **Inclusion criteria**
2. Patients aged 20-80 years
3. Patients ≥ grade B lumbar central canal stenosis who agree to undergo one to two segment posterior spinal decompression surgery
4. Those who are willing to participate and fully comply with the 1-year follow-up protocol
5. Those who have signed an informed consent form (ICF) after he or she (if a signature is available) or a legally authorized representative (LAR) fully understood the contents of the clinical trial
6. **Exclusion criteria**
7. Patients with spondylolisthesis (Meyer grade ≥ II)
8. A history of lumbar spinal surgery at the same level as would be performed during the trial
9. Degenerative lumbar scoliosis (Cobb angle > 20°)
10. Cause of the lumbar spinal stenosis is not of degenerative nature or it is caused by lumbar disc herniation
11. Presence of other spinal disorders in the lesions of the lumbar spinal stenosis (e.g. ankylosing spondylitis, spinal tumor, spinal fracture)
12. Psychological disorders (such as dementia, intellectual disability, or drug abuse)
13. Those who have refused to participate in the study
14. Patients who are deemed unfit for the trial by their physicians

Target Sample Size and Rationale for the Sample Size Calculation

**#** **Patients with lumbar spinal stenosis who will undergo uniportal endoscopic decompressive laminectomy**

- Target sample size: n=120 (Study group: 60, Control group: 60, including Dropout rate at 20 % )
- Primary endpoint: ODI (Oswestry disability index)
- According to a report of existing research [1], the MCID (minimal clinical important difference) of ODI was 12.8, and in another previous study [3], the standard deviation of the ODI value at 1 year after decompressive laminectomy was 18.8. Assuming an equivalence limit of 12.8, under the conditions of alpha = 0.05, power = 0.90, two-sided 95% confidence interval, and follow-up loss at 20%, 60 participants are needed for each group.


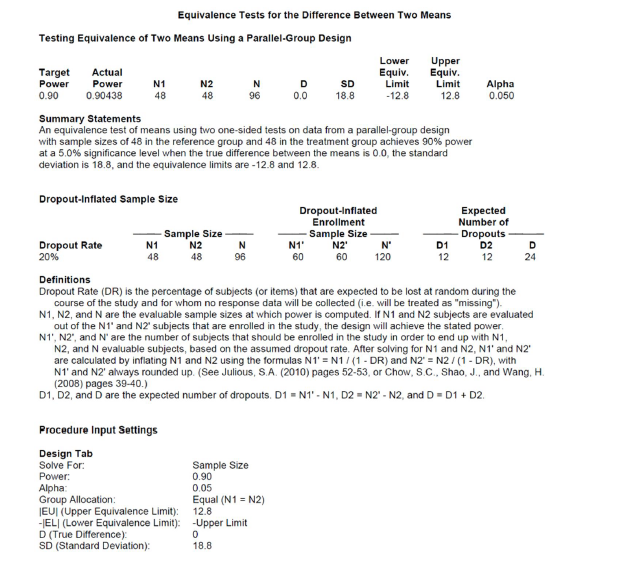


Recruitment of Study Subjects

This study is a prospective, multicenter, clinical trial and subjects will be recruited from Department of Neurosurgery of Seoul St Mary's Hospital, Chungdam Wooridul Spine Hospital, and Wiltse Memorial Hospital (Anyang) of South Korea. Patients who plan to undergo one-to-two segment posterior spinal decompression surgery for lumbar spinal stenosis are recruited as the study subjects and a separate notice for patient recruitment is not used for this trial.

The surgical method investigated in this study is the most commonly used technique for lumbar spinal stenosis, and the clinical study is conducted through pre- and post-operative surveys and analysis of clinical and imaging data, so there is no harm to the subjects. However, if the subject refuses to participate in the study, the trial will not proceed with the subject. In addition, although this study is conducted on a patient group, there is no obligation or undue influence on its participation, and there is no disadvantage to the patient for not participating in the study. Among those who voluntarily agreed to participate in this study and signed an informed consent form (ICF), those who met the inclusion criteria for this study and were not applicable to the exclusion criteria were selected, and subjects were randomly assigned to either of the study or control groups. The Principal Investigator (PI) of this study will not exclude subjects who are likely to participate in this study based solely on race or socioeconomic status. As long as the subject is eligible in terms of inclusion/exclusion criteria, the PI will make every effort to encourage the subjects to participate in the study, and inform the subjects of the objectives of this study so that they can properly represent the total of the patients who will undergo the treatment.

1. Study Period

Study Period

Date of IRB Approval – February 8, 2025

1. Methods
2. Procedure of study

- This is a prospective, multicenter, assessor-blind, randomized, controlled trial.

- According to the patient group assigned to each sub-task, the trial is conducted by dividing into an interlaminar full-endoscopic decompressive laminectomy group (group 1) and a conventional open decompressive laminectomy group (group 2).

- Apart from the difference in the surgical technique, the two groups underwent the same treatment and follow-up (day of surgery, postoperative 2 weeks, 3 months, 6 months, 1 year) preoperatively and postoperatively, and variables for radiographic and clinical outcomes of the surgery are recorded to compare the differences between the two groups. Patients scheduled to be enrolled in this study are briefed on the objectives and methods of the clinical trial from the investigators prior to the study participation and enrolled in the study by signing the informed consent form (ICF). The screening assignment number consists of a series of six-digit numbers. The first digit of the randomization number represents the study institution, the second digit indicates the sub-task, the third digit is S for Screening, and the remaining three digits indicate the serial number for enrollment (Example: B1S-001 -> First Screening Enrollment Patient for Surgery 1 at Seoul National University Bundang Hospital).

- Among the subjects who voluntarily gave their consent to participate in the study and signed the ICF, those who are eligible according to the inclusion and exclusion criteria are randomly assigned to one of the two groups at a ratio of 1:1

The permuted block randomization method is used for randomization. Randomization is applied in sequence from subject number 1 using iCReaT, a web-based eCRF. The investigator will perform the surgery according to the results of the randomization of the subjects. To minimize possible bias that may arise, randomization is performed by a researcher from the Department of Neurosurgery, and the surgeon is informed of the randomization code immediately before surgery.

- The randomization number consists of a series of five-digit numbers. The first digit of the randomization number represents the study institution, the second digit indicates the sub-task, and the remaining three digits indicate the serial number for enrollment (Example: B1-001 -> First Enrolled Patient for Surgery 1 at Seoul National University Bundang Hospital).
- All basic examinations and surveys will be conducted for evaluation before surgery, on the day of surgery, and at follow-up at 2 weeks, 3 months, 6 months, and 1 year postoperatively. The baseline assessment is conducted for the subjects such as baseline radiographic examination, physical examination (age, sex) and medical hearing (past medical history, surgical history) and baseline blood tests (CBC, Routine chemistry).

At baseline and during the preoperative hospitalization period (X-ray, MRI or CT), postoperative 2 weeks, 3 months, 6 months, and 12 months (X-rays), radiographic examinations are performed to examine the status of occurrence of complications. The baseline assessment is performed on ODI, EQ5D, and VAS and postoperative assessment is performed on VAS, ODI, EQ5D, walking, satisfaction, postoperative scarring (POSAS) to evaluate clinical outcomes. Immediately after the surgery, other surgery-related outcomes (postoperative drainage, operative time, duration of hospital stay, postoperative day 1 creatine kinase (CK, CPK)) are measured for comparison of the surgery-related items.

- However, if the subject is unable to pay a visit at the scheduled time due to force majeure reasons (e.g., safety issues due to Covid 19 pandemic), non-face-to-face treatment or telephone surveys allowed by the current law may be applied instead of the actual outpatient visit.

- **Surgical method**

1) Interlaminar full-endoscopic decompressive laminectomy

As shown in the figure below, decompressive laminectomy is performed using endoscopic instruments and instruments for spinal surgery. The method can minimize the injury to normal tissue. After making a skin incision of about 1 cm, the endoscope is introduced, the location is checked on the X-ray, and the radiofrequency electrode is applied to control bleeding. After exposing the lamina, a direction-variable drill for endoscopy is used to perform laminectomy while minimizing the injury of the facet joint to expose the ligamentum flavum. During the process of ligamentum flavum detachment, the inferior edge of cranial lamina and superior margin of the caudal lamina are carefully removed if necessary for additional decompression and to secure operation field for improved visualization.


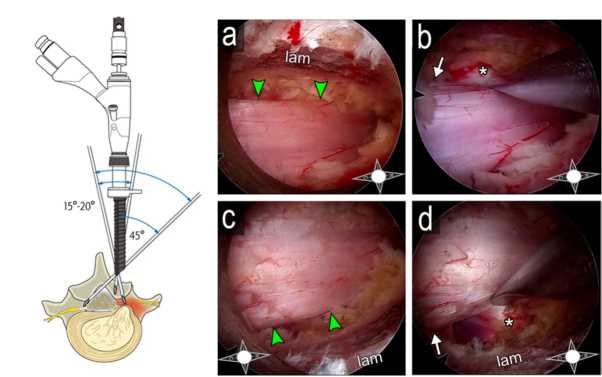


1. **Conventional open decompressive laminectomy**

This method is the commonly applied technique of laminectomy in which a midline incision is made and the bilateral hypertrophied ligamentum flavum is removed with open bilateral access. After disinfection of the surgical site, the surgical site is located with a simple radiograph of the lateral view for the lumbar spine. A skin incision is performed at a length of about 3 cm from the midline of the surgical site to the long axis, and this is sufficient to secure the operational field. After detaching the paraspinal muscles from the spinous process, lamina, and vertebral joint, the fascial flap is opened and the operational field is secured using a retractor.

In the intervertebral foramen, the proximal ligamentum flavum is clearly visible in the transverse process of the upper vertebrae and the distal face of the pedicle. The ligamentum flavum detachment of the target area is carefully performed not to injure the nerve root running below the distal end of the transverse process of the upper vertebrae. During ligamentum flavum detachment, the inferior edge of the cranial lamina and the superior margin of the caudal lamina are carefully removed for additional decompression and securing the operational field. At this time, care must be taken not to damage the facet joint. Thereafter, the nerve root is viewed and decompression is performed by checking the nerve running to the proximal part of the pedicle of the lower vertebrae for sufficient decompression along the nerve root to the distal end. After decompression is performed on the contralateral side in the same way, it is checked whether the bilateral running of the nerve root is without restriction. Then, hemostasis is performed at the surgical site, the surgical site is sutured and disinfected to complete the surgery.


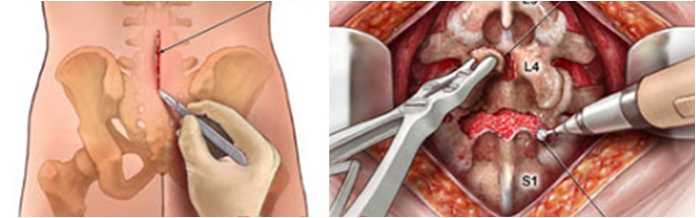


1. Setting of control groups and randomization method

This study will be conducted by dividing the patients with lumbar spinal stenosis who will undergo posterior spinal decompression surgery into the interlaminar full-endoscopic laminectomy group (group 1) and conventional surgery group (group 2) through a prospective RCT. Interlaminar full-endoscopic laminectomy is currently the preferred minimally invasive surgery method. Unilateral laminectomy bilateral decompression (ULBD) is a minimally invasive surgical method without endoscopy, and the clinical outcomes have been reported to show no significant difference from those of conventional surgical techniques. Therefore, it is necessary to verify the equivalence in clinical outcomes between the interlaminar full-endoscopic laminectomy and the conventional open decompressive laminectomy.

This study is a prospective study, and among the subjects who voluntarily gave their consent to participate in the study and signed the ICF, those who are eligible according to the inclusion and exclusion criteria will be randomized to one of the two groups at a ratio of 1:1. The permuted block randomization method is used for randomization. Randomization is applied in sequence from subject number 1 using iCReaT, a web-based eCRF. The investigator will perform the surgery according to the results of randomization of the subjects. To minimize possible bias that may arise, randomization is performed by a researcher from the Department of Neurosurgery, and the surgeon is informed of the randomization code immediately before surgery.

This study has limitations in applying the double-blind method. It is inevitable that the investigator knows which procedure was used, and the patient also knows the surgical method due to the incision site after surgery. Therefore, the assessor-blind method, which applies the blinding only to the assessor, is applied, and an assessor is a third person who has not performed the surgery.

SPIRIT checklist for study schedule

| **Visit Type** | **Screening** | **Operation/**  **Treatment** | **Follow-up** | | | |
| --- | --- | --- | --- | --- | --- | --- |
| **Visit** | **1** | **2** | **3** | **4** | **5** | **6** |
| **Visit week** | **-4~0weeks** | **0 day** | **2 weeks** | **12 weeks** | **24 weeks** | **52 weeks** |
|  |  |  | **± 5 days** | **± 4 weeks** | **± 8 weeks** | **± 8 weeks** |
| Informed consent form (ICF) | ■ |  |  |  |  |  |
| Demographic investigation | ■ |  |  |  |  |  |
| Investigation of medical history and medication history^[1]^ | ■ |  |  |  |  |  |
| Physical examination | ■ |  |  |  |  |  |
| Laboratory tests^[2]^ | ■ |  |  |  |  |  |
| Checking and confirmation of inclusion/exclusion criteria | ■ | ■ |  |  |  |  |
| Randomization |  | ■ |  |  |  |  |
| Surgery |  | ■ |  |  |  |  |
| MRI or CT^[3]^ | ■ | ■ |  |  |  |  |
| X-ray (Radiography)^[4]^ | ■ |  | ■ | ■ | ■ | ■ |
| ODI measurement | ■ |  | ■ | ■ | ■ | ■ |
| EQ5D measurement | ■ |  | ■ | ■ | ■ | ■ |
| VAS measurement | ■ |  | ■ | ■ | ■ | ■ |
| POSAS measurement |  |  |  | ■ | ■ | ■ |
| Other metrics[5] |  |  | ■ | ■ | ■ | ■ |
| Examination of AEs |  | ■ | ■ | ■ | ■ | ■ |

* Visit 1 and visit 2 may proceed concurrently.

1) Investigation of medical history within 3 years from the time of screening (5 years in case of cancer)

2) Laboratory tests:

- Hematology (CBC): WBC, Hb, Hct, ESR - Biochemical profile (routine chemistry): hs-CRP, CPK

1. MRI or CT is performed to measure and examine the degree of canal release and the presence of complications within the hospitalization period after surgery.
2. Simple radiography(X-ray): Simple radiographs will be obtained in the anteroposterior (AP), lateral, lateral-flexion, and lateral-extension views. In order to prevent undergoing excessive radiographic examinations, it is possible to replace the examination results with those taken within 4 weeks from the date of screening or follow-up visit.
3. Other measures: satisfaction with the surgery, returning to daily activities, patient report on walking distance/time, etc.
4. Items of assessment, clinical examination and methods of assessment

**Demographic information:** Age, sex and date of surgery of the study subjects

**Physical examination and laboratory tests:** Medical history, smoking status, blood tests, physical examination

**Radiography:** The subjects undergo simple radiography and MRI (or CT) examination for baseline assessment. After surgery, during the hospitalization period, the degree of canal release, complications, and recurrence are examined through simple radiography and MRI (or CT) scans.

**Oswestry disability index (ODI) measurement:** Scoring of the Oswestry disability index (10 items) is conducted at the visit. The investigator hands out the subject a questionnaire, and asks the subject to rate each item on a five-point scale for self-report. The rated score is expressed as a percentage of the total score of the answered questions excluding the questions not completed by the subject.

**EQ5D-5L measurement:** The EQ5D-5L survey (5 items) is conducted at the visit. The investigator hands out the subject a questionnaire and asks the subjects to rate and indicate each item for self-report. The total score of the indicated items is converted using the EQ5D value set for calculation.

**VAS score (Pain scale):** The degree of pain felt by the subject during activity is assessed using a 100mm VAS (Visual Analogue Scale) for both low back and legs. The investigator explains the VAS to the subject and asks the subject to rate each item and indicate accordingly for self-report. In addition, the degree of pain in the surgical site (Postoperative Day 1 or Day 2) is also investigated using VAS.

**Walking distance and time:** The changes in values in the questions on walking ​​at postoperative 2, 12, 24, and 52 weeks are compared to the baseline values.

**Satisfaction after surgery:** At the 1-year follow-up visit, the investigator hands out the questionnaire to the subject and asks the subjects to indicate for self-report.

**Returning to daily activities postoperatively:** Time required to return to daily activities postoperatively (week)

**POSAS[Patient and Observer Scar Assessment Scale] (patient scale 2.0):** The patient assesses the postoperative scarring with 6 items and scored on a ten-point system, with a score of 1 indicative of normal skin and a score of 10 indicative of the worst scar imaginable, and the total score of the assessment is 60.

**Other surgery-related outcomes**: The size of the surgical skin incision, postoperative drainage, operative time, duration of hospitalization (hours), postoperative Day 1 creatine kinase (CK, CPK) are also evaluated.

**Presence of adverse events and complications:** Adverse events requiring revision surgery related to the intervention. When a patient arrives at the ward after completing the surgery, a resident (or fellow, clinical instructor, nurse, investigator, etc.) who is blinded to the surgical method examines the patient to determine whether there are any complications related to the intervention or any serious complications that require revision surgery.

Primary endpoint

The Primary endpoint of this trial is the difference between the ODI score measured at baseline and that measured 12 months postoperatively**.**

Secondary endpoints

The Secondary endpoints for this trial include clinical outcomes (VAS, EQ5D-5L, ODI, walking distance and time, satisfaction after surgery, time required to return to daily activities postoperatively, POSAS), radiographic outcomes, and other surgery-related outcomes**.**

1. Expected Adverse Events and Precautions

* Expected adverse events(AEs)/risks and countermeasures

The posterior spinal decompression surgery to be performed in this trial is a standard treatment for patients with lumbar spinal stenosis. There are no additional complications specifically from this study other than those related to general spinal surgery.

However, if AEs related to this clinical study occur, the investigator will take the following actions:

When a physician identifies AEs, immediately report to IRB

The physician will proceed with necessary treatment for the AEs

If the subject requires emergency actions due to this trial, the necessary emergency measures shall be taken as soon as possible. In addition, in the case of serious adverse events (SAEs), prompt and appropriate actions shall be taken to minimize possible damage

However, general complications expected after lumbar spinal surgery are not considered as complications “caused by this trial.”

1. Discontinuation and Dropout Criteria

Discontinuation and Dropout Criteria

The principal investigator may consider discontinuation of the clinical study in the following cases.

1. In the event of serious adverse events (SAEs)
2. Other cases where the investigator deems that continuation of the study is difficult

The cases for consideration of subject dropout are as follows.

(1) Non-compliance to the instructions of the investigator

(2) If the subject or LAR withdraws the consent to participate (presented explicit wishes to refuse the participation in the study)

1. Violation of the inclusion/exclusion criteria
2. In the event of serious adverse events SAEs/adverse device effect (ADE)
3. Other cases where the investigator deems that continuation of the study is difficult

In the event of a withdrawal or dropout of a subject, the investigator must make the utmost effort to follow-up the study subject who has withdrawn or dropped out for any reason, and must perform a follow-up monitoring evaluation for safety.

1. Endpoints, Evaluation and Analysis Methods (Statistical analysis method)

Primary endpoint

**The primary endpoint of this trial is the difference between the ODI score measured at baseline and that measured 12 months postoperatively.**

Statistical analysis method Interlaminar full-endoscopic laminectomy will be considered equivalent to open decompressive laminectomy with regard to the surgical outcome if the upper and lower limits of the 95% confidence interval (CI) of the treatment difference value of each group’s ODI score at 12 months postoperatively fall within the pre-defined equivalence margin (+-12.8).

Secondary endpoints

The secondary endpoints for this trial include clinical outcomes (VAS, EQ5D-5L, ODI, walking distance and time, satisfaction after surgery, time required to return to daily activities postoperatively, POSAS), radiographic outcomes, and other surgery-related outcomes.

**VAS, EQ5D-5L, ODI, walking distance and time**

Postoperative changes in the scores of the clinical outcomes against the baseline scores at each time point.

Statistical analysis method : For each group, the differences from the baseline values to the postoperative values and differences in the pattern of change between the two groups are analyzed using repeated measures analysis of variance (ANOVA). In addition, for specific within-group or between-group comparisons at each time point, the t-test is used, while applying the adjusted significance level considering the multiplicity of the test.

**Satisfaction after surgery, time required to return to daily activities postoperatively, Patient and Observer Scar Assessment Scale(POSAS scale)**

Statistical analysis method: The satisfaction after surgery, the time to return to daily activities postoperatively, and POSAS scale score at the time of final postoperative follow-up are comparatively analyzed between the two groups using the t-test.

**Radiographic outcomes**

Examination of the occurrence of complications during the final follow-up using simple radiographs (Development of spondylolisthesis, progression of degenerative lumbar spinal conditions, etc.), analysis of surgical outcomes through postoperative MRI (or CT)

Statistical analysis method: The occurrence of complications is examined for each group, and Chi- square test or t-test is used for comparative analysis on the difference between the two groups for the degree of central canal release and the canal dimension measured using postoperative MRI or CT.

**Other surgery-related outcomes**

Comparison between the two groups on other surgery-related outcomes. [size of the surgical skin incision, postoperative drainage, operative time, duration of hospitalization (hours), Postoperative (From immediately after surgery to discharge from the hospital), postoperative Day 1 creatine kinase (CK, CPK)]

Statistical analysis method: The degree of difference between the two groups is evaluated using the chi-square test or t-test.

General statistical analysis method

**General principles of analysis**

- For continuous variables, descriptive statistics (number of subjects, mean, standard deviation, median, minimum, maximum) are presented, and for categorical variables, frequency (N) and percentage (%) are presented.
- In the analysis of the primary efficacy endpoint, if missing data occurs due to dropout, etc., the data are processed as a failure and the non-responder impuatation (NRI) method is used.
- If the clinical trial is discontinued according to the discontinuation criteria, the data are handled as missing values.
- In the analysis of secondary efficacy endpoints, when there are missing values in the data, a method of last observation carried forward (LOCF) is used.
- For the analysis of the safety set, when there are missing values in the data, the data shall be analyzed as it is without performing imputation for the missing values (OC method).

**Handling of analysis sets**

- The analysis sets are categorized into safety set, full analysis set (FAS) and per protocol (PP) set.
- Efficacy data are analyzed using both FAS and PP, and final determination on efficacy endpoints is conducted by FAS analysis, whch is the analysis based on the modified ITT (intention to treat).
- Data on safety are analyzed for the safety sets.
- For the analysis of efficacy endpoints, when there are missing values in the data, the data shall be analyzed as it is without performing imputation for the missing values.

**Definition of analysis sets**

- FAS (Full Analysis Set): Subjects who met the inclusion/exclusion criteria and who underwent randomization to receive the intervention at least once during the trial period are included in FAS. In the efficacy analysis, regardless of the actual status of undergoing the surgery, analysis is performed based on the treatment group assigned by randomization.
- PP (Per Protocol Set): Subjects who have completed the trial without major protocol violations among the full analysis set (FAS) are included in PP set. However, if the trial is discontinued according to the discontinuation criteria, the clinical trial is considered to be completed and included in the PP analysis. For items of major protocol violations, see Section 13-5.
- Safety Set: Subjects who were randomized and have undergone the surgery at least once during the trial period are included in the safety set. In the safety analysis, analysis is performed based on the classification according to the actual type of surgery received.

1. Evaluation and Reporting of Safety including Adverse Events

Adverse events occurring during the clinical trial are followed up until the symptoms are resolved or stabilized. Adverse events that occur are coded using Preferred Terms (PTs) according to the Medical Dictionary for regulatory activities (MED-DRA). All adverse events (AEs) and serious adverse events (SAEs) that occurred after treatment and the unexpected adverse events (UAEs) related to the treatment in the trial are summarized according to the severity by the time point of onset using Preferred Terms (PTs) and prepared in graphs. These are recorded based on the self-report of the subjects and examination during their visits. In recording the AE-related information, the name and duration, the scope and severity of symptoms, the causal relation with the surgical procedure, additional treatment, the outcome of AEs, and the status of seriousness are described in detail in the Special Form of the case report form (CRF).

Statistical analysis method: In this study, AEs indicate cases of new or worsened symptoms or signs that were not observed during the baseline assessment and include all signs, symptoms and diseases regardless of the causal relation with the surgical procedure. In the event of AEs, the name of the related symptoms, time of onset, duration, severity of symptoms, and causal relation with the surgical procedure are recorded in CRF. The number of cases and the number of applicable subjects are calculated for each case of AEs, and the rate of occurrence of adverse events 95% two-sided confidence intervals are presented. The difference in the rate of AE occurrence between the two groups is compared using the chi-square test or Fisher's exact test.

1. Protocol on patient damage compensation

See “Attachment 4”

AEs caused by participation in this study will be processed according to the provisions on the “Protocol on Patient Damage Compensation”.

1. Post-study Treatment of Subject and Treatment Criteria

After the termination of the clinical study, continuous follow-up monitoring and observation are performed according to the usual standard treatment schedule. For side effects and AEs that occurred after the clinical study was completed, if the causal relationships with the investigational surgical method falls under “definitely related”, “probably related”, or “possibly related”, the patient will be compensated according to the procedure specified in “Protocol on Patient Damage Compensation”. The criteria for the causal relation are based on the severity of the AEs, the evaluation method of the association (-Mild: the presence of subjective or objective symptoms, but the AE not interfering with daily activity. - Moderate: Some interference with daily activity. a decrease in the dose of concomitant medications or treatment is required because of the AE. - Severe: Prevents daily activity. Life threatening or death), and causal relation (Definite, Probable, Possible, Unlikely, Unclassified, Unknown).

After the termination of the clinical trial, the patient will be diagnosed through observation during additional follow-up period.

1. Safety protection measures for the subjects
2. Safety protection measures for the subjects

1) Ethical compliance

This study is conducted based on the 2013 WMA Declaration of Helsinki with regard to the rights and welfare of the subjects. The principal investigator or investigator shall provide an explanation of the purpose and all possible consequences of this study and the trial will be conducted for those subjects who signed the informed consent form (ICF) indicating that the subject will voluntarily participate in the study.

The team of the investigators related to this clinical study shall familiarize themselves with the study protocol with accuracy, and the principal investigator shall take all necessary precautions such as adequate response to the occurrence of unexpected AEs, required reporting, and education/training for the participating investigators, and the trial is conducted based on the guidelines for Good Clinical Practice by ICH (ICH-GCP).

2) Procedure of consent by subject

“Informed Consent Form (ICF)” is a document representing voluntary consent of the study subject or legally authorized representative (LAR) of the subject to participate in the clinical trial. The ICF shall be signed with the free will of the subject in an environment completely excluded from external pressure. The content and nature of the proposed clinical trial, the anticipated risks and potential benefits, and the requirements for the trial shall be explained in detail so that the applicable subject can fully understand the content to make an informed decision. The investigator shall provide an explanation of his trial for around 5 minutes to the prospective study participant. The waiting time between the study briefing process and the consent process is set to be 24 hours.

An ICF must be obtained in accordance with the guidelines of the regional institutional review board ( IRB) prior to the initiation of any trial-related procedures (i.e. any procedures described in the protocol). The procedure for obtaining the consent and signing the ICF shall be recorded in the supporting document of the subject, and a signed and dated copy of the ICF shall be kept together.

The subject or his/her LAR cannot read, an impartial witness must be present throughout the ICF consent process. An impartial witness is defined as a person who is not involved in the conduct of the clinical trial at all, is not affected by the personnel of the clinical trial, and participates in the subject consent process to read the ICF and other consent-related documents such as patient information leaflet to the subject. If the ICF is provided for the subject, the subject or the LAR of the subject reads the form, all questions raised by the subject/LAR are answered, and the subject or the subject's LAR verbally gives consent to the subject's participation in the study, if possible, the subject or the subject's LAR must sign and date the ICF in person, and the impartial witness must also sign and date the ICF. By signing the ICF, it signifies that the impartial witness confirms that the contents of the ICF and other consent-related documents have been accurately explained to the subject or the LAR of the subject, that they have fully understood, and that the subject or the LAR of the subject has voluntarily given their consent.

Since there is no age limit for the study subjects, if it is deemed necessary for the elderly subjects aged 75 years or older, the consent of the subject and his/her LAR will be obtained together in the consent process.

1. Data and Safety Monitoring Plan

1) Monitoring personnel

*Monitoring manager: Prof. Jin-Sung Kim, Department of Neurosurgery,

Seoul St Mary’s Hospital, The Catholic University of Korea

*Monitoring staff: Won-Seok Seo, Helptrial PM, Ji-Wuk Bong, CRM

2) Items of study accruals and safety data monitoring

***Items of study accruals: Questionnaire used in the trial, e-CRF, ICF, EMR**

*** Safety items: EMR, e-CRF, adverse events, serious adverse events**

1. Method and period of study data and safety monitoring

In order to monitor and confirm whether clinical trials are conducted in accordance with the KGCP and related regulations and in accordance with the study protocol, monitoring staff delegated by the monitoring manager shall perform the monitoring. Both the on-Site monitoring, which is conducted by visiting the stud institution in person, and in-house monitoring through the EDC system are performed.

On-site monitoring will be conducted a total of 10 times, including the site initiation visit and site close out visit.

The integrity and accuracy of the data collected by Helptrial's monitor (e-CRF) are monitored, and the dataset of the e-CRF system built by Helptrial is used. For data with problems or that need clarifications, the queries are sent to the investigator through the query function of the e-CRF system for the investigator to look into the data and make necessary corrections. 15/17

1. Reporting of AEs, protocol observance issues and other unexpected problems

Monitoring and auditing are carried out to ensure that clinical study is conducted in accordance with KCGP guidelines and data can be accepted for registration at home and abroad. The scope of monitoring for this clinical trial is EMR (evidence document), follow-up data on protocol violation cases, interim report, observation and records for cases of AEs, and all other materials related to the trial including all records of the investigator files. AEs and unexpected problems identified during monitoring, deviation from the protocol, AEs and unexpected problems related to death or life will be reported in compliance with relevant laws and IRB regulations.

1. Withdrawal of Study

The principal investigator (PI) may suspend part or all of the trial if it is deemed not reasonable to continue the clinical study in light of the outcomes observed during the course of this study. When PI has prematurely terminated or suspended this study, this shall be immediately notified to IRB and a detailed report on the reasons for the premature termination or suspension shall be submitted. When the IRB has terminated or suspended approval for this study, the PI shall prepare and submit a detailed report on the reasons for the premature termination or suspension. If the study is prematurely terminated or suspended, PI shall immediately inform the subject of this information so that appropriate actions and follow-up can be carried out.

1. Confidentiality of Clinical Study Documents and Subject Records

Confidentiality of the subject information must be protected. For all study-related records, the patient's EMR number and hospital registration number shall be kept in separate files under the responsibility of the PI, and they shall be coded for de-identification of personal information in the study data. Alternatively, the study data shall be saved in a password-protected file and stored in a locked laboratory. Each subject is assigned a number with an identification code number for de-identification of personal information, and the name is recorded with initials. The PI must ensure the confidentiality of all information related to this study. According to Article 15 of the Enforcement Rules of the Bioethics and Safety Act, the study-related records must be kept for 5 years from the time point of study termination, and when the data retention period is over, they must be destroyed according to Article 16 of the Enforcement Decree of the Personal Information Protection Act. However, if the plan for retention of records is changed for follow-up trial, records, data accumulation, etc., the records are retained for a longer period. The information of this study can only be disclosed to IRB or related investigators. PI may only use the results of this study for the purposes of registration, publication, and information provision for medical and pharmaceutical experts.

1. Other matters necessary for safe and scientifically sound clinical study

Completion of the clinical trial training for the team of investigators

List of Attachments: Please attach all applicable documents from the following to the online system.

- A sample patient information leaflet/informed consent form (ICF)
- A template for the case report form
- Two reference papers

□ Others (If applicable, please submit the following documents in addition: Notice of patient recruitment, protocol on patient damage compensation and related insurance policy, notice on medical care benefits confirmation, a letter of approval from MFDS (Ministry of Food and Drug Safety), etc.
