# Supplementary material for: Evaluation of the efficacy and safety of conventional and interlaminar full-endoscopic decompressive laminectomy to treat lumbar spinal stenosis (ENDO-F trial): Protocol for a prospective, randomized, multicenter trial
Source: PLoS One. 2023 Apr 5;18(4):e0283924. doi: 10.1371/journal.pone.0283924 (PMC10075401; doi:10.1371/journal.pone.0283924)
Supplement: S3 File — (PDF) [file pone.0283924.s003.pdf]

임상연구계획서

(기타 의학적 중재(수술, 방사선치료 등)를 포함하는 연구용)

1. 연구 과제명

국문: **요추부 협착증에서 후궁간 경유 경피적 단일공 내시경하 후방감압술과 고식적 후방 감압술의 임상적 및 방사선학적 유효성 및 안전성 평가 (ENDO-F Trial): 전향적, 무작위 배정, 평가자 눈가림, 다기관 연구**

영문: Evaluation of the efficacy and safety of conventional and interlaminar full-endoscopic decompressive laminectomy to treat lumbar spinal stenosis (ENDO-F Trial): A prospective, randomized, Assessor blind, multicenter trial

2. 임상연구 실시기관명 및 주소

기관명	주소
가톨릭대학교 서울성모병원	서울시 서초구 반포동 반포대로 222, 서울성모병원 신경외과
우리들병원	서울시 강남구 청담동 47-4
윌스기념병원 (안양)	안양시 동안구 경수대로 560(호계동)

3. 연구자

	서울성모병원	우리들병원	윌스기념병원 (안양)
책임연구자	김진성 교수 **(세부책임자)**	배준석 병원장	이동찬 병원장
공동연구자	임우정 임상강사<br>김정훈 임상강사	신상하 원장<br>은상수 원장<br>금한중 원장<br>최용수 원장	김태현 원장<br>신승호 원장<br>홍현진 원장<br>김지연 원장
연구담당자	김은 연구원<br>이초롱 연구원	이윤주 연구원	김도연 연구원

4. 의뢰자(기관)명 및 주소

가톨릭대학교 서울성모병원 신경외과 김진성

5. 연구 목적

척추관 협착증에서 내시경 수술 방법이 기존 고식적 수술과 비교하여 **임상적 결과의 동등성을 확인해 보고자 한다.** 또한 내시경 수술의 전향적 임상데이터를 분석하여 **임상 진료 지침에 대한 근거를** 마련하고, 치료법 선택에 대한 임상 진료 지침을 제시하고자 한다. 이를 위해 총 3 개 기관에서 요추 협착증 환자에서 단일공 내시경을 이용한 후방 감압술의 **임상적 효용성 및 안정성을** 기존의 고식적 수술법과 비교해 보고자 한다.

6. 연구 배경

본 연구에서 대상으로 하는 척추 질환 환자는 건강심사평가원에 따르면 2018년 한 해에만 약 363만명으로 보고되었으며 이는 5년간 45만명이 늘어난 수치임. 특히 척추관 협착증의 경 우 5년 새 약 32.4% 늘어난 수치이다. 국민건강보험 2018년 수술 통계 연보에 따르면 내시경 하 척추 수술은 7,218건으로 전년도 5,108건에 비해 41.3% 증가하였으며, 그로 인한 진료비 역시 45.9% 증가하였음. 같은 기간 고식적 척추 수술 건수가 165,573건에서 169,706건으로 2.4% 증가한 것에 비하여 중대한 증가를 확인할 수 있어 척추 내시경 수술의 확장세가 두드러지고 있다고 할 수 있다. 또한 노인 인구의 증가에 따른 척추 질환의 유병률은 점차 증가될 것으로 보이며, 의료 비용의 부담도 같이 늘어날 것으로 예상된다.

요추 협착증에서의 후방 감압술 (Posterior decompression)과 요추 추간판 탈출증에서의 후방 접근 추간판 절제술 (discectomy)은 환자의 증상을 해결하기 위해 사용되고 있는 가장 고식적인 방법이다. 기존 고식적 방법은, 출혈이 많고, 수술 후 통증, 불안정성, 척추 주변 근육의 감소 등 문제가 있어 해부학적 구조물을 보존하기 위한 최소 침습 수술을 시행한다. 최소 침습수술의 대표적인 방법으로 편측 접근 후방 감압술 방법 (Unilateral laminectomy Bilateral decompression, ULBD)이 가장 많이 사용되고 있으며, 극상돌기 절개를 통한 방법, 내시경을 이용한 방법 등이 이용되고 있다. 최소 침습 수술은 기존 고식적 방법에 비해 많은 장점들이 있으며 임상적 결과도 기존 고식적 방법과 차이가 없다고 보고되어 있다.

최근 **내시경을 이용한 감압술과 추간판 절제술이 개발**되어 사용되고 있다. 내시경을 이용한 척추 수술에는 기구가 들어가는 개방공의 개수에 따라 단일공 내시경 수술법과 양방향 내시경 수술법으로 나뉜다. 1cm 이내의 피부 절개창을 통하여 수술부위에 접근하기 때문에 정상 구조물의 손상을 최소화 할 수 있어 수술 후 통증, 신경 유착 등의 합병증이 적다.

그러나 내시경을 이용한 척추 수술은 **후향적 연구로는 임상적 결과가 좋다고 보고된 바 있으나 다기관, 전향적, 무작위 배정 연구로는 아직 그 임상적 결과가 확인되지 않았다.** 또한 현재 요추 협착증 환자에서 시행하는 고식적 수술과 미세 현미경 수술은 보험 급여 항목으로 인정을 받아 수술이 필요한 환자들에게 적절하게 사용되고 있는 반면에, 척추 내시경 수술은 국내에서 아직 적절하게 인정받지 못하여 일부 추간판 탈출증 환자에서만 제한적으로 시행되고 있다. 국내에서 보고된 척추 내시경 수술 연구의 대부분은 후향적 연구이며 고식적 수술에 비한 내시경 **수술의 효용성과 안정성을 확립하기 위한 level 1 study는 부족하다.** 따라서 본 연구에서는 다기관 전향적 무작위 배정 연구를 통해 내시경 수술의 효용성과 안정성을 임상적 결과가 확립된 고식적 후방 감압술의 임상결과와 비교해 보고자 한다.

7. 연구 대상

요추 중심관 협착증 Grade가 B 이상으로 1-2 분절 후방 감압술을 시행하기로 한 사람

1년 이상 추시 가능한 사람

8. 연구대상자의 선정기준, 제외기준, 목표한 대상자의 수 및 그 근거

선정 및 제외기준

# 요추부 협착증 환자

(1) 선정기준

A. 만 20세부터 80세

B. 요추 중심관 협착증 Grade가 B 이상으로 1-2 분절 후방 감압술을 시행하기로 한 사람

C. 1년 이상 추시 가능한 사람

D. 본인(서명이 가능한 경우에 한함) 또는 법적 대리인이 임상시험의 내용을 충분히 이해한 후 동의서에 서명한 연구 대상자

(2) 제외기준

A. 척추체 전위가 50% 이상인 척추전방전위증

B. 이전에 같은 분절 수술 시행한 사람

C. 요추 퇴행성 측만증 (Cobb angle > 20도)

D. 협착증의 원인이 퇴행성이 아니거나 추간판탈출증에 의한 경우

E. 요추 협착증 이환부위에 다른 척추 질환이 있는 경우 (강직성 척추염, 종양, 압박골절 등)

F. 정신과적 질환 (치매, 정신지체자, 심한 약물 중독 등)

G. 연구에 참여하기 거부한 사람

H. 기타 임상시험 담당자가 임상시험 대상으로 부적당하다고 판단하는 환자

목표한 대상자 수 및 산출 근거

# 요추부 협착증 환자에서 단일공 내시경을 이용한 후방 감압술

- 목표 연구 대상자수: 120명 (시험군: 60명, 대조군: 60명, 탈락율 20 % 포함)
- Primary outcome: ODI (Oswestry disability index)
- 기존 논문[1]에 의하면 ODI의 MCID (Minimal clinical important difference)는 12.8 이었으며, 이전 연구[3]에서 decompressive laminectomy 수술 후 1년째 ODI 값의 standard deviation은 18.8이었다. 동등성 한계를 12.8로 정하고 alpha = 0.05, power = 0.90, two-sided 95% confidence interval, follow-up loss = 20%로 가정하면 각 군당 60명이 필요하다.

Equivalence Tests for the Difference Between Two Means

Testing Equivalence of Two Means Using a Parallel-Group Design

Target Power	Actual Power	N1	N2	N	D	SD	Lower Equiv. Limit	Upper Equiv. Limit	Alpha
0.90	0.90438	48	48	96	0.0	18.8	-12.8	12.8	0.050

Summary Statements

An equivalence test of means using two one-sided tests on data from a parallel-group design with sample sizes of 48 in the reference group and 48 in the treatment group achieves 90% power at a 5.0% significance level when the true difference between the means is 0.0, the standard deviation is 18.8, and the equivalence limits are -12.8 and 12.8.

Dropout-Inflated Sample Size

	Sample Size			Dropout-Inflated Enrollment Sample Size			Expected Number of Dropouts		
Dropout Rate	N1	N2	N	N1'	N2'	N'	D1	D2	D
20%	48	48	96	60	60	120	12	12	24

Definitions

Dropout Rate (DR) is the percentage of subjects (or items) that are expected to be lost at random during the course of the study and for whom no response data will be collected (i.e. will be treated as "missing").

N1, N2, and N are the evaluable sample sizes at which power is computed. If N1 and N2 subjects are evaluated out of the N1' and N2' subjects that are enrolled in the study, the design will achieve the stated power.

N1', N2', and N' are the number of subjects that should be enrolled in the study in order to end up with N1, N2, and N evaluable subjects, based on the assumed dropout rate. After solving for N1 and N2, N1' and N2' are calculated by inflating N1 and N2 using the formulas N1' = N1 / (1 - DR) and N2' = N2 / (1 - DR), with N1' and N2' always rounded up. (See Julious, S.A. (2010) pages 52-53, or Chow, S.C., Shao, J., and Wang, H. (2008) pages 39-40.)

D1, D2, and D are the expected number of dropouts. D1 = N1' - N1, D2 = N2' - N2, and D = D1 + D2.

Procedure Input Settings

Design Tab

Solve For:	Sample Size
Power:	0.90
Alpha:	0.05
Group Allocation:	Equal (N1 = N2)
|EU| (Upper Equivalence Limit):	12.8
-|EL| (Lower Equivalence Limit):	-Upper Limit
D (True Difference):	0
SD (Standard Deviation):	18.8

연구 대상자 모집 계획

본 연구는 전향적, 다기관 연구로써, 서울성모병원 신경외과, 우리들병원, 윌스기념병원 (안양)에서 대상자를 모집할 계획이다. 요추부 척추관 협착증으로 한 분절 혹은 두 분절 후방 감압술을 시행하기로 한 사람을 대상으로 연구 대상자를 모집하며 연구대상자 모집 공고문은 사용하지 않는다.

본 연구는 척추관 협착증에서 가장 흔히 사용되는 수술 방법으로 수술 전후 설문과 임상적 및 영상학적 분석을 통해 이루어지는 연구이므로 연구대상자에게 피해가 가는 부분은 없다. 하지만 연구대상자가 연구에 참여하기를 거부한다면 시행하지 않는다. 또한 이 연구가 환자군을 대상으로 진행하는 연구이지만 이에 대한 강제성이나 부당한 영향은 전혀 없으며 연구에 참여하지 않는다 하여 불리한 부분은 없다. 본 연구에 자의적으로 참여할 것을 동의하고 동의서에 서명한 자 중 본 연구의 선정기준에 합당하며 제외기준에 포함되지 않은 자를 선정하고 대상자는 시험군 및 대조군 중 한 군에 각각 무작위로 배정될 것이다. 본 연구의 책임연구자는 인종이나 사회경제적 상태에만 근거해서 이 연구에 참여할 가능성이 있는 연구대상자를 배제시키지 않을 것이다. 이 연구의 선정기준에 합당하다면, 가능한 연구대상자들이 이 연구에 참여할 수 있도록 모든 노력을 다할 것이며 본 기관에서 치료받는 연구대상자의 전체를 대표할 수 있도록 연구대상자들에게 연구의 목적을 주지시킬 것이다.

9. 연구 기간

연구기간

IRB 승인일 ~ 2025년 02월 28일

10. 연구 방법

1) 구체적인 연구방법

- 다기관, 평가자 눈가림, 무작위배정, 대조군 비교, 전향적 임상시험으로 전향적, 무작위 배정 연구를 시행한다.
- 각 세부 과제에 배정된 환자 군에 따라 내시경군 (1군)과 고식적 수술군 (2군)으로 나누어 연구를 시행하고자 한다.
- 수술 방법의 차이 외에 두 군은 수술 전 후 동일한 처치와 추시관찰 (수술일, 수술 후 2주, 3개월, 6개월, 1년)을 하면서 방사선학적, 임상적 수술 결과에 대한 변수를 기록하여 두 군간의 차이를 비교한다. 이 연구에 등록 예정인 환자는 연구에 참여하기 전에 연구 담당자로부터 연구에 대한 목적, 방법을 듣고 동의서에 서명함으로써 연구에 등록이 된다. 스크리닝 배정 번호는 일련의 여섯 자리 번호로 부여되며 무작위 배정번호의 첫째 자리는 임상시험실시기관을 의미하고, 둘째 자리는 세부과제를 의미하며, 세번째 자리는 Screening의 S, 나머지 세 자리는 등록의 일련번호를 의미한다. (예시: B1S-001 -> 분당서울대병원의 1세부 수술의 첫 스크리닝 등록 환자)
- 연구에 자의적으로 참여할 것을 동의하고 동의서에 서명한 연구대상자 중 포함 및 제외 기준을 만족하는 연구대상자는 두 군 중 한 군에 1:1의 비율로 각각 무작위로 배정된다.
- 무작위 배정은 permuted block randomization 방법을 이용한다. 무작위 배정은 Web-based e-CRF인 iCReaT을 이용하여 연구대상자 번호 1번부터 순차적으로 적용한다. 연구자는 연구대상자의 무작위 배정 결과에 따라 해당 수술을 시행할 예정이다. Bias를 최소화하기 위해 randomization은 연구원이 시행을 하며, 수술 직전 무작위배정 코드를 수술자에게 통보한다.
- 무작위 배정 번호는 일련의 다섯자리 번호로 부여되며 무작위 배정번호의 첫째 자리는 임상시험실시기관을 의미하고, 둘째 자리는 세부과제를 의미하며, 나머지 세 자리는 등록의 일련번호를 의미한다. (예시: B1-001 -> 분당서울대병원의 1세부 수술의 첫 등록 환자)
- 모든 기본적인 검진이나 설문을 수술 전과 수술, 수술 후 2주, 3개월, 6개월 및 1년 추시시 평가한다. 수술 전 이 연구대상자들을 대상으로 수술 전 방사선학적 평가, 임상적 신체검진 (나이, 성별) 및 병력청취 (과거력, 수술력)를 시행하고, 수술 전 검사상 혈액검사 (CBC, Routine chemistry)로 확인한다. 수술 전과 수술 후 입원기간 중에 (X-ray, MRI 혹은 CT), 수술 후 2주, 3개월, 6개월, 12개월 영상의학적 검사 (X-ray)를 통해 합병증 유무를 확인한다. 수술 전 ODI, EQ5D, VAS, 수술 후 VAS, ODI, EQ5D, 보행, 만족도, 흉터설문(POSAS)를 설문조사 하여 임상적 평가를 시행한다. 수술 직후 기타 수술과 관련된 항목 (수술 후 출혈량, 수술 시간, 입원 기간, 수술 후 1일 째 Creatine Kinase (CK, CPK))을 측정하여 수술과 관련된 항목을 비교한다.

- 단, 불가항력적인 이유 (예를 들면 Covid 19로 인한 안전상의 이슈 등)로 예정된 시기에 방문하지 못할 경우 현행법이 허락하는 비대면 진료 혹은 전화 설문으로 대신할 수 있다

- **수술 방법**

1) 후궁간 경유 경피적 단일공 후방 감압술

아래 그림과 같이 내시경 기구와 척추 수술기구를 이용하여 후방 감압술을 시행한다. 정상 조직의 손상을 최소화 할 수 있다. 1cm 정도의 피부절개를 한 후 내시경을 삽입한 후 엑스레이상 위치를 확인 한 후 고주파를 이용하여 지혈을 하고 후궁을 노출한 후 내시경용 드릴을 이용하여 후관절의 손상을 최소화 하면서 후궁절제를 시행한 후 황색인대가 노출되도록 한다. 황색인대 절제술을 시행하면서 추가적인 감압 및 시야 확보를 위해 필요한 경우 상부 척추의 후궁 뼈 아래 부분과 하부 척추의 후궁 뼈 윗부분을 조심스럽게 제거한다.

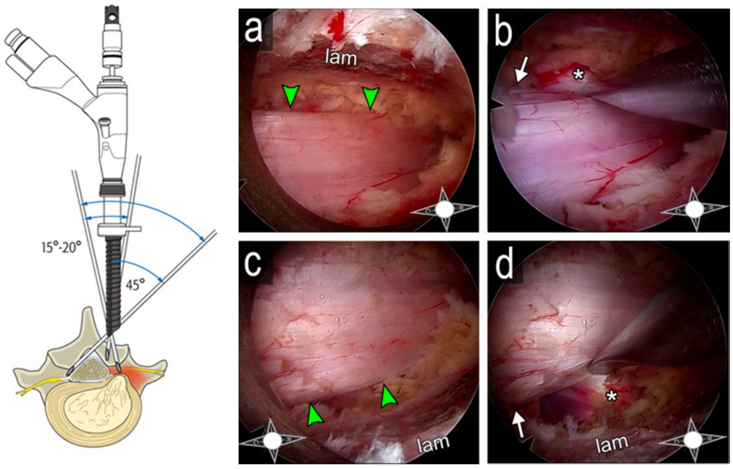

2) 고식적 후방 절제술

정중 절개선을 넣고 개방적으로 양측으로 접근하여 양측의 비후된 황색인대를 제거하는 방법으로서 현재 보편적인 후궁 절제술 방법이다. 수술 부위 소독 이후, 요추 측위 단순 방사선 촬영으로 수술 부위를 확인한다. 수술 부위의 정중선에서 장축으로 약 3 cm 길이로 피부 절개를 시행하며, 이 정도로 충분히 시야를 확보할 수 있다. 척추측방근육을 극상돌기, 후궁 및 척추관절로부터 박리 하여 근막피판을 젖힌 후 견인기를 이용하여 수술 시야를 확보한다.

추간공에서 황색인대 근위부는 상부 척추 횡돌기와 척추경(pedicle) 원위부 표면에서 확실하게 구분된다. 목표 구획의 황색인대 절제술을 시행할 때는 상부 척추 횡돌기의 원위부 말단 아래로 주행하는 신경근을 손상시키지 않도록 주의해야 한다. 황색인대 절제술을 시행하면서 추가적인 감압 및 시야 확보를 위해 상부 척추의 후궁 뼈 아래 부분과 하부 척추의 후궁 뼈 윗부분을 조심스럽게 제거하게 되는데, 이때 후관절이 손상받지 않도록 주의해야 한다. 이후 신경근이 확인하고, 신경근의 주행을 따라 말단부까지 충분히 감압을 하기 위해 하부 척추의 척추경 근위부까지 신경주행을 확인하며 감압을 시행한다. 반대쪽도 같은 방법으로 감압을 시행한 후 양측 신경근 주행이 자유로운지 확인한다. 이후 수술부위 지혈을 하고 수술부위 봉합 및 소독을 한 뒤 수술을 마친다.

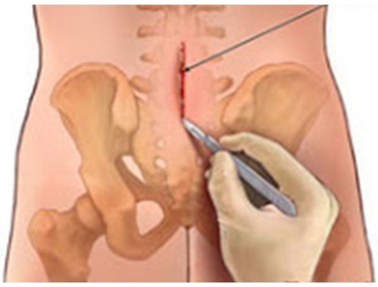

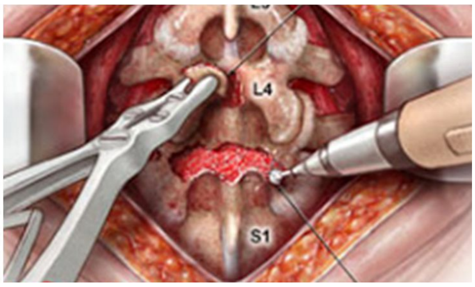

2) 비교군 설정 및 무작위 배정 방법

본 연구는 전향적, 무작위 배정 연구를 통해 요추 협착증에 대한 후방 감압술을 시행하는 환자에서 후궁간 경유 경피적 단일공 내시경군 (1군)과 고식적 수술군 (2군)으로 나누어 연구를 시행하고자 한다. 후궁간 경유 경피적 단일공 내시경군은 최근 최소침습적 수술방법으로 선호되고 있는 방법이다. 편측 접근 수술법은 내시경을 이용하지 않은 최소침습적 수술 방법이며, 임상적 결과는 고식적인 수술법에 비해 차이가 없다. 따라서 최소침습적 수술방법과 표준 수술방법인 고식적 수술법과 임상적 결과를 비교하여 차이가 없음을 증명하는 것이 필요하다.

본 연구는 전향적 연구로서, 연구에 자의적으로 참여할 것을 동의하고 동의서에 서명한 연구대상자 중 포함 및 제외 기준을 만족하는 연구대상자는 두 군 중 한 군에 1:1의 비율로 각각 무작위로 배정된다. 무작위 배정은 permuted block randomization 방법을 이용한다. 무작위 배정은 Web based eCRF인 iCReaT을 이용하여 연구대상자 번호 1번부터 순차적으로 적용한다. 연구자는 연구대상자의 무작위 배정 결과에 따라 해당 수술을 시행할 예정이다. Bias를 최소화하기 위해 randomization은 신경외과 연구원이 시행을 하며, 수술 직전 무작위배정 코드를 수술자에게 통보한다.

이 연구는 이중 맹검법을 적용하기에는 한계가 있다. 연구자 스스로 어떠한 술식을 사용했는지 알게 되며, 환자는 수술 상처를 확인해서 수술 방법을 알게 된다. 따라서 평가자에게만 눈가림 방법을 적용하는 평가자 맹검법을 적용하며, 임상 관찰자는 수술을 시행하지 않은 제3자로 한다.

연구수행일정표

Visit Type	Screening	Operation/ Treatment	Follow-up			
Visit	1	2	3	4	5	6
Visit week	-4~0weeks	0 day	2 weeks	12 weeks	24 weeks	52 weeks
			± 5 days	± 4 weeks	± 8 weeks	± 8 weeks
연구대상자 서면동의	■					
인구학적정보조사	■					
병력/수술력조사[1]	■					
신체검사	■					
실험실적 검사[2]	■					
선정/제외기준 확인	■	■				
무작위배정		■				
수술		■				
MRI 혹은 CT[3]	■	■				
X-ray 검사[4]	■		■	■	■	■
ODI 설문	■		■	■	■	■
EQ5D 설문	■		■	■	■	■
VAS 설문	■		■	■	■	■
POSAS 설문				■	■	■
기타 설문[5]			■	■	■	■
이상사례 수집		■	■	■	■	■

* 방문1과 방문2는 동시에 진행이 가능함.

1) 스크리닝 시점 3년 이내 과거 병력 조사(단, 암인 경우 5년 이내)

2) 실험실적 검사:

- 혈액학적: WBC, Hb, Hct, ESR
- 혈액생화학적: hs-CRP, CPK

3) 수술 후 입원기간 내에 감압의 정도, 합병증 유무를 관찰하기 위해 MRI 또는 CT를 시행한다.

4) 단순방사선 검사(X-ray): 전후방(AP), 측방(Lateral), 측방 굴곡(Lateral-flexion), 측방 신전(Lateral-extension)을 측정한다. 과도한 검사가 진행되는 것을 방지하기 위해 스크리닝일 또는 추적방문일로부터 4주 이내 검사한 결과가 있다면 갈음 가능하다.

5) 기타설문: 수술만족도, 일상생활, 보행설문 등

11. 관찰항목, 임상검사항목 및 관찰검사방법

인구학적 정보: 연구대상자의 나이, 성별, 수술일

신체검진 및 기본검사: 기왕력, 흡연, 기본 혈액검사, 신체검사

방사선 검사: 연구대상자는 수술 전 단순 방사선 검사 및 MRI (혹은 CT) 검사를 시행한다. 수술 후 입원기간 중에 단순 방사선 검사 및 MRI (혹은 CT) 촬영을 통해 감압 정도, 합병증 발생 유무, 재발 여부를 확인한다.

Oswestry disability index (ODI) 설문: 방문 시 Oswestry disability index 설문(10개 문항)을 실시한다. 연구담당자가 연구 대상자에게 설문지를 주고 각 문항마다 0-5점의 점수가 부과된 답변을 연구 대상자가 스스로 평가하여 표시하 도록 한다. 연구대상자가 작성하지 않은 문항은 제외하고 표시한 문항의 총 점수 대비 평가 점수를 백분율로 계산한다

EQ5D-5L 설문: 방문 시 EQ5D-5L 설문(5개 문항)을 실시한다. 연구담당자가 연구대상자에게 설문지를 주고 연구대상 자가 스스로 평가하여 표시하도록 한다. 표시한 문항의 합계 점수를 EQ5D value set을 이용하여 변환하여 계산한다.

VAS score (통증 척도): 연구 대상자가 활동 시 느끼는 통증의 정도를 허리, 다리 두 영역에 대해 100mm VAS(Visual Analogue Scale)를 이용하여 검사한다. 연구담당자가 연구대상자에게 VAS에 대해 설명하고 연구대상자가 스스로 평가하여 표시하도록 한다. 또한 수술 부위 통증(수술 후 1일, 2일)에 대해서도 VAS를 이용하여 통증 정도를 조사한다.

보행가능 시간: 수술 전 대비 수술 후 2, 12, 24, 52주 보행 설문 값의 변화를 확인한다.

수술 후 만족도: 1년 추시 방문 시. 연구담당자가 연구대상자에게 설문지를 주고 연구대상 자가 스스로 평가하여 표시하도록 한다.

수술 후 일상생활로의 복귀: 수술 후 일상생활로 복귀까지 걸리는 시간(주)

수술 후 흉터 (POSAS[Patient and Observer Scar Assessment Scale] patient scale 2.0): 수술 후 흉터에 대해서 환자가 6가지 항목으로 평가하며 1~10까지 점수 중 정상이 1점, 가장 안 좋을수록 10점이며 총점 60점으로 계산

기타 수술과 관련된 항목: 수술 incision크기, 수술 후 출혈량 (drain), 수술 시간, 입원 기간 (시간), 수술 후 1일째 Creatine Kinase (CK, CPK)를 체크한다.

이상반응, 합병증 유무: 수술과 관련된 재수술을 요하는 adverse event, 환자가 수술을 끝내고 병동에 도착하면 수술 방법에 대해 blind 되어있는 전공의(또는 전임의, 임상강사, 간호사, 연구원 등)가 수술 관련 합병증 또는 재수술을 요하는 중대 합병증이 있는지 관찰

주 평가변수

본 연구의 1차 평가 변수는 수술 후 1년째 추시 관찰 시 ODI 값의 차이이다.

부 평가변수

본 연구의 2차 평가 변수는 임상적 평가 (VAS, EQ5D-5L, ODI, 보행가능 시간, 수술 후 만족도, 일상생활복귀 기간, POSAS), 영상의학적 평가, 기타 수술과 관련된 항목이다.

12. 예측 부작용 및 사용상의 주의사항

*** 예측되는 부작용/위험 및 대처 방안**

본 연구에서 시행할 요추 후방 감압술은 요추부 척추관 협착증 치료로서 일반적으로 시행하는 표준 치료법으로, 일반적인 척추 수술에 대한 합병증 이외에 본 연구로 인해서 추가되는 합병증은 없다.

하지만, 만약 본 연구와 관련된 부작용 발생시 연구 담당자는 다음과 같은 조치를 취한다.

① 의사는 부작용을 발견하였을 경우, 즉시 IRB에 신고한다.

② 의사는 필요한 의학적 조치를 취한다.

본 연구로 인해서 연구 대상자에게 피해가 발생하여 응급조치가 필요한 경우, 가능한 빨리 응급조치를 시행한다. 또한 중대한 유해사례 발생 시 빠르고 적절한 조치를 취하여 가능한 피해를 최소화할 것이다.

단. 일반적인 요추 수술 후 예상되는 합병증의 경우는 "본 연구로 인한" 합병증으로 간주하지 않는다.

13. 중지 및 탈락 기준

중지·탈락 기준

다음의 경우 연구책임자는 임상연구의 중지를 고려할 수 있다.

(1) 연구대상자에게 중대한 이상반응이 일어난 경우

(2) 기타 책임 연구자의 판단에 임상의 지속이 곤란하다고 판단되는 경우

다음의 경우 연구책임자는 연구대상자의 탈락을 고려할 수 있다.

(1) 연구대상자가 연구자의 지시에 불응하는 경우

(2) 연구대상자(또는 법적 대리인)이 동의를 철회한 경우(임상연구 거부 의사를 제시함)

(3) 선정/제외 기준을 위반한 경우

(4) 심각한 이상반응/이상의료기기 반응이 발생한 경우

(5) 책임 연구자의 판단에 임상연구 지속이 곤란하다고 판단되는 경우

책임연구자는 연구대상자가 중지 또는 탈락한 경우 어떠한 사유든지 중지 또는 탈락한 시험 대상자를 추적관찰하기 위한 모든 노력을 기울이며, 안전성에 대한 추적관찰 평가를 수행해야 한다..

14. 효과 평가기준, 평가방법 및 해석방법 (통계분석방법)

주 평가변수

본 연구의 1차 평가 변수는 수술 후 1년째 추시 관찰 시 ODI 값의 차이이다.

통계적 분석 방법: 수술 후 1년째 각 군의 ODI값의 treatment difference 값의 95% 신뢰구간이 Equivalence margin 마진 (+- 12.8)에 들어올 경우 (포함될 경우) 동등하다고 판단한다.

부 평가변수

본 연구의 2차 평가 변수는 임상적 평가 (VAS, EQ5D, 보행가능시간, 수술 후 만족도, 수술 후 일상생활복귀 기간, POSAS), 영상의학적 평가, 기타 수술과 관련된 항목이다.

VAS, EQ5D-5L, ODI, 보행가능 시간

수술 전 대비 수술 후 기간별 임상적 평가 점수의 변화양상

통계적 분석 방법 : 각 군 별로 수술 전 대비 수술 후, 수술 전후 차이와 양 군 간 변화양상의 차이를 repeated measures ANOVA로 분석한다. 또한 수술 후 각 시점에서 집단 내 또는 집단간 차 이의 구체적인 비교는 t-test로 비교하되, 검정의 다중성을 고려하여 조정된 유의수준을 적용한다.

수술 후 만족도, 수술 후 일상생활복귀 기간, 수술 후 흉터 (POSAS scale)

통계적 분석 방법: 수술 후 최종 추적 관찰 당시의 수술 후 만족 정도와 수술 후 일상생활복귀기간, POSAS scale score를 t-test를 이용하여 양 군 간 비교 평가한다.

영상 검사

단순 방사선 검사상 최종 추시시 합병증 유무 (전방전위증 발생, 퇴행성 변화의 진행 등), 수술 후MRI (혹은 CT)를 통한 수술 결과 분석

통계적 분석 방법: 각 군 별로 합병증 발생 유무를 확인, 수술 후 MRI 혹은 CT를 통한 추간판 절제 정도를 Chi-square test 혹은 t-test를 이용하여 양 군 간 차이의 정도를 평가한다.

수술과 관련된 기타 항목

수술과 관련된 기타 항목에 대한 양군간 비교. [수술 incision크기, 수술 후 출혈량, 수술 시간, 입원 기간 (시간), 수술 후 (수술 후 ~퇴원시), 수술 후 1일째 Creatine Kinase (CK, CPK)]

통계적 분석 방법: 양 군간 차이를 Chi-square test 혹은 t-test를 이용하여 양 군 간 차이의 정도를 평가한다.

일반적인 통계방법

결과분석의 일반적 원칙

· 연속형 변수의 경우에는 기술 통계량(시험대상자 수, 평균, 표준편차, 중앙값, 최소값, 최대값)을 제시하고, 범주형 변수에 대해서는 빈도(N) 및 비율(%)을 제시

· 일차 유효성 분석 시, 중도 탈락등의 사유로 결측치가 발생하는 경우 해당 데이터는 실패로 간주하여 처리하는 NRI (non-responder impuation) 방법을 사용

· 중지기준에 따라 임상시험이 중지된 경우는 결측치 처리하고

· 이차 유효성 분석 시 결측치가 발생 했을시에는 LOCF 방법을 사용

· Safety set 분석은 결측치 보정 없이 관측치 그대로(OC method) 사용

분석군의 처리

· Safety set, FAS (full analysis set)와 PP (per protocol)로 나눔

· 유효성에 대한 자료는 FAS 및 PP를 모두 사용하여 분석하며, 유효성 평가변수에 대한 최종판정은 modified ITT(intention to treat)인 FAS 분석으로 실시
· 안전성에 대한 자료는 Safety set을 대상으로 분석
· 유효성 평가 자료 중 결측치가 발생한 경우에는 수치를 대체하지 않고 결측 처리

분석군의 정의

· FAS (Full Analysis Set) 대상군: 선정/제외 기준에 적합하였고, 무작위 배정되어 시험기간 동안 한 번이라도 수술받은 시험 대상자를 대상으로 한다. 유효성 분석 시에는 실제 수술과 상관없이 무작위 배정받은 치료군으로 포함하여 분석한다.
· PP (Per Protocol Set) 대상군: FAS 분석군 중에서 중대한 시험계획서 위반 없이 시험을 완료한 시험 대상자를 대상으로 한다. 단 중지기준에 따라 임상시험이 중지된 경우는 임상시험을 완료한 것으로 보고 PP분석에 포함한다. 중대한 임상시험계획서 위반에 대한 항목은 13-5절을 참고한다.
· 안전성 (Safety Set) 대상군: 무작위 배정되어 시험기간 동안 한 번이라도 수술받은 시험 대상자를 대상으로 한다. 안전성 분석 시에는 실제로 수술받은 치료군으로 포함하여 분석한다.

15. 부작용을 포함한 안전성의 평가기준, 평가방법 및 보고방법

임상연구기간 동안 발생된 이상반응은 증상이 해결될 때까지 또는 안정될 때까지 추적 관찰한다. 발생되는 이상반응은 MED-DRA(Medical Dictionary for regulatory activities)을 이용한 선호용어로 코드화한다. 치료 후 발생한 모든 이상반응(AEs), 중대한 이상반응(SAEs), 임상연구 치료와 관련된 예상하지 못한 이상반응(UAEs)을 중증도에 따라 시작 시점별로 선호용어를 이용하여 요약하고 도표로 작성하며, 시험대상자의 자발적인 보고 및 내원 시 확인하여 기록한다. 기록시 명칭과 지속기간, 증상의 범위, 정도, 의료기기와의 인과관계, 추가적인 치료, 이상반응의 결과, 중대성 등에 대하여 증례기록서의 Special Form에 구체적으로 기술한다.

통계적 분석 방법: 본 연구에서 이상반응은 이식 수술 전에는 관찰되지 않았던 증상 또는 징후가 수술 후 새로 나타나거나 악화된 경우로서, 기기와의 관련성에 무관하게 나타난 모든 증후(sign), 증상(symptom), 질병을 포함한다. 이상반응이 나타나면 관련 증상명, 발현시기, 지속기간, 증상의 정도 및 시험기기와의 인과관계 등에 대하여 증례기록서에 기록하고 발생한 이상반응에 대해서는 각 이상반응 별로 건수와 해당 연구 대상자 수를 계산하며, 이상반응 발현율과 95% 양측 신뢰구간을 제시한다. 양 군간 이상반응률의 차이는 카이제곱검정 또는 Fisher의 exact test로 비교한다.

16. 피해자 보상에 대한 규약

별첨4” 참고

본 연구참여로 발생한 이상반응에 대하여 피해자 보상규약에 따라 처리할 것임.

17. 임상연구 후 대상자의 진료 및 치료기준

임상연구 종료 후에도 통상적인 표준 치료 일정에 따라 지속적인 추시 및 관찰을 시행한다. 임상연구가 종료된 후 발생한 부작용 및 이상반응 등에 대하여 임상연구용 수술방법과 인과관계가 "관련성이 명백함", "관련성이 많음", "관련성이 의심됨"에 해당하는 경우 피해자 보상 절차에 따라 보상절차가 진행된다. 관련성에 대한 기준은 이상반응의 중증도, 관련성 평가방법 (- 경증: 자각적 또는 타각적 증상은 있으나 일상생활에는 지장이 없다.

- 중등증: 일상생활에 지장을 느낄 정도의 증상. 이상사례 때문에 병용약물의 감량 또는 치료를 필요로 하는 정도
- 중증: 일상생활을 제대로 할 수 없는 정도의 증상. 생명위협 또는 사망), 인과관계 (확실함, 상당히 확실함, 가능함, 가능성 적음, 평가곤란, 평가불가)에 근거한다.

임상시험 종료 후 추가 추시 기간의 관찰을 통해 진단받게 된다.

18. 대상자의 안전보호 대책

1. 연구대상자의 안전보호를 위한 대책

1) 연구의 윤리성 확보를 위한 기본 방안

본 연구는 2013년 헬싱키 선언에 입각하여 연구대상자의 권리와 복지를 염두에 두고 준비된 것으로 연구책임자 또는 담당자는 본 연구의 목적 및 모든 가능성에 대해 설명하고 연구대상자 자신이 자발적으로 연구에 참여하겠다는 연구대상자동의서에 서명한 연구대상자를 대상으로 한다.

본 연구와 관련된 연구진은 연구계획을 정확히 숙지하도록 하고, 연구책임자는 예상하지 못한 이상반응의 출현에 대한 충분한 대처와 필요한 보고, 연구 참여진에 대한 교육 등 사전조치를 취하며, 연구의 진행은 ICHGCP 기준에 따라 진행하도록 한다.

2) 연구대상자의 동의 과정

"임상시험대상자 동의서"란 임상시험대상자 또는 임상시험대상자의 법적 보호자가 임상시험에 참여한다는 자발적 동의를 표한 문서이다. 동의서는 외압이 철저히 배제된 환경에서 자유 의지로 작성되어야 한다. 당사자가 충분히 이해할 수 있도록 제안된 임상시험의 내용과 성격, 예상되는 위험과 잠재적 혜택, 임상시험 요건을 자세히 설명하여 충분한 정보를 토대로 결정을 내릴 수 있도록 해야 한다. 책임 연구자는 잠재적 연구 참

여자에게 본 연구에 대해 설명을 5분여에 걸쳐 시행을 한다. 연구 설명 과정과 동의 취득 과정 사이에 대기 시간은 24시간이다.

어떠한 연구 관련 절차(즉, 계획서에 기술된 모든 절차)도 시작하기 전에 지역 IRB 지침에 따라 연구대상자 동의서를 반드시 취득해야 한다. 연구대상자 동의서 취득 절차는 연구대상자 근거문서에 기록해야 하며 서명 및 기일된 동의서 사본을 함께 보관해야 한다.

연구대상자 또는 연구대상자의 법적 보호자가 글을 읽을 수 없다면, 연구대상자 동의서 취득 절차 내내 공정한 참관인이 동석해야 한다. 공정한 참관인이란 연구 수행에 전혀 관여하지 않고 연구 관계자의 영향을 전혀 받지 않으며, 연구대상자 동의 절차에 참여하여 연구대상자 동의서와 기타 동의 설명서 문서를 연구대상자에게 읽어줄 사람으로 정의된다. 연구대상자에게 연구대상자 동의서를 제공하고, 이를 연구대상자 또는 연구대상자의 법적 보호자가 읽고 궁금한 점에 대해 답변을 듣고, 연구대상자 또는 연구대상자의 법적 보호자가 연구대상자의 연구 참여를 구두로 동의하면, 가능한 경우 연구대상자 또는 연구대상자의 법적 보호자가 동의서에 직접 서명 및 기일하고 공정한 참관인도 동의서에 직접 서명 및 기일해야 한다. 공정한 참관인은 동의서에 서명함으로써 동의서와 기타 동의설명서의 내용을 연구대상자 또는 연구대상자의 법적 보호자에게 정확하게 설명하였고 그들이 충분히 이해하였고 연구대상자 또는 연구대상자의 법적 보호자가 자유 의지로 동의를 표했음을 인정하는 것이다.

연구 대상자의 연령제한이 없으므로 만 75세 이상의 노인 대상자에서는 필요하다고 판단되는 경우 대상자 및 대상자의 대리인의 동의를 같이 취득하여 진행할 것이다.

2. 자료 및 안전성 모니터링 계획 (Data and Safety Monitoring Plan)

1) 모니터링 책임자

***모니터링 책임자: 가톨릭대학교 서울성모병원 신경외과 김진성 교수**

***모니터링 담당자: 헬프트라이알 PM 서원석, CRM 봉지욱**

2) 자료 및 안전성 정보 모니터링 항목

***자료(study accruals) 항목: 임상 설문지, e-CRF, 동의서, EMR**

***안전성(Safety) 항목: EMR, e-CRF, 이상사례, 중대한 이상사례**

3) 자료 및 안전성 모니터링 방법 및 주기

임상시험이 KGCP 및 관련규정에 따라 그리고 임상시험계획서에 따라 실시되는지의 여부를 확인하기 위해서 모니터링 책임자의 위임을 받은 모니터링 담당자에 의하여 수행한다. 모니터링은 기관을 직접 방문하여 실시

하는 On-Site Monitoring과 EDC 시스템을 통한 In-House Monitoring을 병행하여 실시한다

On-Site 모니터링은 총 10 회에 걸쳐 진행될 예정이며, Site Initiation Visit과 Site Close Out Visit은 포함하여 실시한다

헬프트라이얼의 모니터가 수집한 자료(e-CRF)의 완전성과 정확성에 대해 모니터링하고, 헬프트라이얼에서 구축한 e-CRF 시스템의 데이터세트를 이용하며, 문제가 있거나 의문이 있는 자료에 대해서는 e-CRF 시스템의 쿼리 기능을 통하여 연구자에게 보내어 확인하고 자료를 수정한다.

4) 이상반응보고, 연구 미준수, 예상하지 못한 문제의 보고

임상 연구가 KCGP 기준에 따라 실시되고 자료가 국내외에서 등록 시 인정될 수 있도록 하기 위해 모니터링 및 감사를 실시한다. 본 연구의 모니터링 범위는 EMR(근거문서), 계획서 위반 사례 F/u, 중간보고, 이상반응 사례 관찰 및 기록, 연구자 파일의 모든 기록 외 연구와 관련된 제반 사항이다. 모니터링 시 발견된 이상반응 및 예상하지 못한 문제, 계획서 미준수 사항, 사망이나 생명과 연관된 이상 반응이나 예상하지 못한 문제 등의 경우는 관련법 및 IRB 규정을 준수하여 보고할 예정이다.

5) 연구 중단 기준

연구책임자는 본 연구 과정에서 관찰된 결과에 비추어 본 연구를 지속하는 것이 현명하지 않다고 판단될 경우 본 연구의 일부 혹은 전부를 중지시킬 수 있다. 연구 중단 기준은 연구책임자가 본 연구를 조기종료 또는 일시 중시시켰을 경우, 책임자는 이 사실을 임상시험심사위원회에게 즉시 알리고, 조기종료 및 일시 중지에 대한 상세한 사유서를 제출하여야 한다. 임상시험심사위원회가 본 연구에 대한 승인을 종료 또는 일시 중지 시켰을 경우, 연구책임자는 조기종료 및 일시 중지에 대한 상세한 사유서를 제출하여야 한다. 연구가 조기종료 또는 일시 중지된 경우 연구책임자는 연구대상자에게 이 사실을 즉시 알리고 적절한 조치와 추적 관찰이 이루어질 수 이루어질 수 있도록 한다.

3. 연구대상자의 개인정보보호 방안

연구대상자 정보에 대하여 비밀이 보장되어야 한다. 연구와 관련된 모든 기록물은 환자의 의무기록번호 및 병록번호는 주관연구자의 책임하에 별도의 파일로 보관하며 이를 코드화하여 연구데이터를 통하여 개인 신상 확인이 불가능하도록 관리한다. 또는 연구데이터는 패스워드가 걸린 파일에 저장하여 잠금 장치가 있는 연구실에 보관하도록 한다. 각 시험대상자의 개인 신상정보가 확인되지 않도록 식별코드 번호로 번호를 부여하며 성명은 이니셜로 기록한다. 연구책임자는 본 연구와 관련하여 모든 정보에 대해 비밀보장을 해야만 한다. 생명윤리법 시행규칙 제 15조에 따라 연구 관련 기록을 연구가 종료된 시점부터 5년간 보관하여야 하며, 보관기간이 지나면 개인정보보호법 시행령 제 16조에 따라 파기하도록 한다. 다만, 후속 연구, 기록, 축적 등을 위해 보관 계획이 변경된다면 추가

보관하도록 한다. 본 연구의 정보는 오직 임상시험심사위원회나 관련된 연구진에게만 개방할 수 있다. 시험책임자는 본 시험의 결과를 등록, 발표 및 의약학 전문가들을 위한 정보제공 등의 목적으로만 사용할 수 있다.
**19. 기타 임상연구를 안전하고 과학적으로 실시하기 위하여 필요한 사항**
연구진의 임상연구 교육 이수
☞ **첨부서류**: 다음 중 해당하는 서류를 전산시스템에 첨부하여 주십시오.<br>□ 대상자 설명문/동의서 양식<br>□ 증례기록서 양식<br>□ 근거논문 2 편<br>□ 기타 (해당 사항 있을 경우, 다음과 같은 서류를 추가로 제출하십시오<br>: 대상자 모집 공고문, 피해자 보상규약 및 관련 보험 증권, 요양급여확인통보서, 식약처 승인서 등
