# Supplementary material for: Evaluation of the efficacy and safety of conventional and interlaminar full-endoscopic decompressive laminectomy to treat lumbar spinal stenosis (ENDO-F trial): Protocol for a prospective, randomized, multicenter trial
Source: PLoS One. 2023 Apr 5;18(4):e0283924. doi: 10.1371/journal.pone.0283924 (PMC10075401; doi:10.1371/journal.pone.0283924)
Supplement: S5 File — (DOCX) [file pone.0283924.s005.docx]

[Form 1-2]

Protocol

**Title (Korean):**

**요추부 협착증에서 후궁간 경유 경피적 단일공 내시경하 후방감압술과 고식적 후방 감압술의 임상적 및 방사선학적 유효성 및 안전성 평가 (ENDO-F Trial): 전향적, 무작위 배정, 평가자 눈가림, 다기관 연구**

Title (English):

**Evaluation of the efficacy and safety of conventional and interlaminar full-endoscopic decompressive laminectomy to treat lumbar spinal stenosis (ENDO-F Trial): A prospective, randomized, Assessor blind, multicenter trial**

**Study Sites**

|  | Seoul St. Mary’s Hospital | Chungdam Wooridul Spine Hospital | Wiltse Memorial Hospital (Anyang) |
| --- | --- | --- | --- |
| Principal Investigator | Prof. Jin-Sung Kim (Coordinating Investigator) | Junseok Bae, President of Hospital | Dong Chan Lee, President of Hospital |
| Sub-investigators | Woojung Lim, Clinical Instructor  Junghoon Kim, Clinical Instructor | Sang-Ha Shin, Director  Sang Soo Eun, Director  Han Joong Keum, Director  Young Soo Choi, Director | Seung Ho Shin, Director  Hyun Jin Hong, Director  Ji Yeon Kim, Direcctor  Tae Hyun Kim, Department Head |
| Study Coordinators | Eun Kim , Researcher  Cho-Rong Lee , Researcher | Yun-Ju Lee , Researcher | Do-Yeon Kim , Researcher |

IRB Number: Approved Date:

Revised Date:

Wiltse Memorial Hospital 994-3, Ingye-dong, Paldal-gu, Suwon

Gyeonggi-do, 442-833, Republic of Korea

TEL: +82-31-240-6000 FAX： +82-31-240-6282

Protocol Abstract

| **Title: Evaluation of the efficacy and safety of conventional and interlaminar full-endoscopic**  **decompressive laminectomy to treat lumbar spinal stenosis (ENDO-F Trial): A prospective,**  **randomized, Assessor blind, multicenter trial** | | | |
| --- | --- | --- | --- |
| **Title: 요추부 협착증에서 후궁간 경유 경피적 단일공 내시경하 후방감압술과 고식적 후방 감압술의 임상적 및 방사선학적 유효성 및 안전성 평가 (ENDO-F Trial): 전향적, 무작위 배정,**  **평가자 눈가림, 다기관 연구** | | | |
| **Investigators:** | | | |
|  | Seoul St. Mary’s Hospital | Chungdam Wooridul Spine Hospital | Wiltse Memorial Hospital (Anyang) |
| Principal Investigator | Prof. Jin-Sung Kim (Coordinating Investigator) | Junseok Bae, President of Hospital | Dong Chan Lee, President of Hospital |
| Sub-investigators | Woojung Lim, Clinical Instructor  Junghoon Kim, Clinical Instructor | Sang-Ha Shin, Director  Sang Soo Eun, Director  Han Joong Keum, Director  Young Soo Choi, Director | Seung Ho Shin, Director  Hyun Jin Hong, Director  Ji Yeon Kim, Direcctor  Tae Hyun Kim, Department Head |
| Study Coordinators | Eun Kim , Researcher  Cho-Rong Lee , Researcher | Yun-Ju Lee , Researcher | Do-Yeon Kim , Researcher |
|  | | | |
| **Research referral agency: Professor Jin-Sung Kim, Department of Neurosurgery, Seoul St. Mary’s Hospital**  **Expected Study Period: IRB approval date – February 28, 2025** | | | |
| Objectives:  **Primary objectives:** The study aims to **verify the equivalence of clinical outcomes** of endoscopic surgery to those of a conventional surgical technique for patients with lumbar spinal stenosis. In addition, the prospective clinical data of endoscopic surgeries will be analyzed to establish the **basis for clinical practice guidelines** and present a guideline for selection of treatment methods in clinical practice.  **Secondary objectives:** To this end, the **clinical efficacy and safety** of posterior decompressive laminectomy using the full-endoscopic uniportal technique will be compared with those of the conventional surgical method in patients with lumbar spinal stenosis at three study sites. | | | |
| **Rationale:**  According to the Health Insurance Review and Assessment Service (HIRA), the number of patients with spinal diseases, the target population of this study, was reported to be around 3.63 million in 2018 alone, showing an increase of 450,000 patients over the past five years. In particular, the number of patients with lumbar spinal stenosis (LSS) increased by approximately 32.4% in 5 years. According to the 2018 Statistical Yearbook of Surgery by the National Health Insurance, there were 7,218 cases of endoscopic spine surgery, an increase of 41.3% from 5,108 cases in the previous year, and the treatment cost also increased by 45.9%. Compared to the increase of 2.4% in the number of conventional spine surgeries from 165,573 cases to 169,706 cases for the same period, a significant increase is confirmed, indicating that the cases of endoscopic spine surgery show a trend of remarkable growth. In addition, the prevalence of spinal diseases is expected to gradually escalate with the increase in the senior population, and the burden of increased medical expenses for the treatment is also projected.  Posterior decompression in lumbar spinal stenosis and posterior lumbar discectomy in lumbar disc herniation are the most conventional methods used to resolve the patient's symptoms. | | | |

The conventional methods have problems in terms of much bleeding, postoperative pain, instability and a decrease of muscles around the spine, and therefore, minimally invasive surgery is performed to preserve anatomical structures. Unilateral laminectomy bilateral decompression (ULBD) is the representative, most commonly used minimally invasive technique, and other methods such as spinous process osteotomy and endoscopy are also used.

It has been reported that minimally invasive surgery has a number of advantages compared to the conventional surgical technique, and the clinical outcomes are not different from those of the conventional open laminectomy. Recently, surgical techniques of spinal decompression and discectomy using endoscopy have been developed and applied in clinical practice. The endoscopic spine surgery is classified into uniportal endoscopic technique and biportal endoscopic technique depending on the number of ports for insertion of instruments. Since the surgical site is accessed through a skin incision of less than 1 cm, damage to the normal structure can be minimized, resulting in fewer postoperative complications such as pain and epidural adhesion.

However, although spinal surgeries **have been reported with good clinical outcomes in retrospective studies, clinical outcomes have not been confirmed in a multicenter, prospective, randomized clinical trial**. In addition, while the conventional open laminectomy and microscopic surgery currently performed on patients with lumbar spinal stenosis are covered by national health insurance and are appropriately applied for patients in need of surgical treatments, endoscopic spine surgeries are not fully accepted for health insurance coverage, and are performed only for limited number of patients with lumbar disc herniation. Most of the endoscopic sinal surgery studies reported in Korea are retrospective studies, and **only a handful of level 1 studies are available for establishing the efficacy and safety** of the endoscopic surgery compared to the conventional open laminectomy. Therefore, in this study, the efficacy and safety of endoscopic surgeries were compared with those of conventional open laminectomy with established clinical outcomes through a multicenter, prospective randomized trial.

Study design:

- Conduct a prospective, randomized study as a multicenter, assessor-blind, randomized, controlled, prospective clinical trial.
- The study will be conducted with the endoscopic group (Group 1) and conventional surgery group (Group 2) depending on the patient group assigned to the sub-study.

- In addition to the difference in surgical method, the two groups will be compared by recording the variables for radiology and clinical surgical outcomes while receiving the identical treatments and follow-up (day of surgery as well as 2 weeks, 3 months, 6 months and 1 year after the surgery). The patients planned to be enrolled in this study will be given an explanation on the objectives and method of the study from the study coordinators before participating, and become enrolled in the study by signing the informed consent form. Screening assignment numbers are assigned as a 6-digit number – the first digit of the randomization number represents the clinical trial site; the second digit represents the sub-study; the third digit represents S in Screening; and the last three digits represent the serial number for enrollment (e.g. B1S-001 -> first screened patient for detailed surgery 1 at Seoul National University Bundang Hospital)

- Among subjects who have agreed to participate in the study voluntarily and have signed the informed consent form, those who meet the inclusion and the exclusion criteria will be randomized to one of the two groups in 1:1 ratio.
- For randomization, the permuted block randomization method will be used. Randomization will be applied to subjects in sequential order starting from Subject no. 1 using iCReaT, a web-based eCRF. The investigator will perform the corresponding surgery depending on the randomization result for each subject. To minimize bias, randomization will be performed by a researcher and the randomization code will be notified to the surgeon immediately prior to the surgery.
- The randomization number is assigned as a series of 5-digit number in which the first digit represents the clinical trial site; the second digit represents the sub-study; and the remaining 3 digits represent the serial number for enrollment (e.g. B1-001 -> first enrolled patient for detailed surgery 1 at Seoul National University Bundang Hospital)
- All basic tests and questionnaires will be conducted at prior to surgery, on the day of surgery as well as at follow-up 2 weeks, 3 months, 6 months and 1 year after the surgery.

Before surgery, conduct pre-surgery radiological assessment, clinical physical examination (age, gender) and medical history taking (disease history, surgical history) for these subjects, and check the results of the clinical laboratory tests (CBC, Routine chemistry). Check for complications based on radiological imaging tests (X-ray) before surgery, immediately after surgery (X-ray, MRI or CT), and 2 weeks, 3 months, 6 months and 12 months after the surgery. Perform clinical assessments by conducting questionnaires on VAS, ODI, EQ5D, gait, satisfaction, and POSAS before surgery and 2 weeks, 3 months, 6 months and 12 months after the surgery. Immdiately after the surgery, take measurements on the items related to the surgery (postsurgery bleeding, duration of surgery, hospital stay, Creatine Kinase (CK, CPK) at postoperative day 1) to compare surgery-related items.

- However, if the subject is unable to visit the site at planned dates due to unavoidable reasons (such as safety issues due to COVID-19), non-face-to-face treatment or telephone surveys permitted by the current laws can replace the actual outpatient visit.

Eligibility:

Patients with ≥ grade B lumbar central canal stenosis who agree to undergo one to two segment posterior spinal decompression surgery, those who can be followed up for at least 1 year.

Inclusion Criteria:

1. Ages 20 to 80
2. Patients ≥ grade B lumbar central canal stenosis who agree to undergo one to two segment posterior spinal decompression surgery
3. Those who can be followed up for at least 1 year
4. Subjects who signed the informed consent form on own (if the subject is able) or through a legally authorized representative after fully understanding the details of the clinical trial

Exclusion Criteria:

1. Patients with spondylolisthesis (Meyer grade ≥ II)
2. Those who have had the same segmental surgery
3. Degenerative lumbar scoliosis (Cobb angle > 20°)
4. Cause of the lumbar spinal stenosis is not of degenerative nature or it is caused by lumbar disc herniation
5. Presence of other spinal disorders in the lesions of the lumbar spinal stenosis (e.g. ankylosing spondylitis, spinal tumor, spinal fracture)
6. Psychological disorders (such as dementia, intellectual disability, or drug abuse)
7. Those who have refused to participate in the study
8. Other patients deemed ineligible by the investigator to participate in the clinical trial

Treatment Plan:

- Surgical Method

1. Interlaminar full-endoscopic decompressive laminectomy

As shown in the figure below, decompressive laminectomy is performed using endoscopic instruments and instruments for spinal surgery. The method can minimize the injury to normal tissue. After making a skin incision of about 1 cm, the endoscope is introduced, the location is checked on the X-ray, and the radiofrequency electrode is applied to control bleeding. After exposing the lamina, a direction-variable drill for endoscopy is used to perform laminectomy while minimizing the injury of the facet joint to expose the ligamentum flavum. During the process of ligamentum flavum detachment, the inferior edge of cranial lamina and superior margin of the caudal lamina are carefully removed if necessary for additional decompression and to secure operation field for improved visualization.


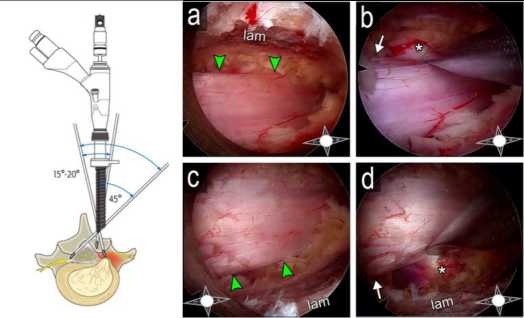


**2) Conventional open decompressive laminectomy**

This method is the commonly applied technique of laminectomy in which a midline incision is made and the bilateral hypertrophied ligamentum flavum is removed with open bilateral access. After disinfection of the surgical site, the surgical site is located with a simple radiograph of the lateral view for the lumbar spine. A skin incision is performed at a length of about 3 cm from the midline of the surgical site to the long axis, and this is sufficient to secure the operational field. After detaching the paraspinal muscles from the spinous process, lamina, and vertebral joint, the fascial flap is opened and the operational field is secured using a retractor.

In the intervertebral foramen, the proximal ligamentum flavum is clearly visible in the transverse process of the upper vertebrae and the distal face of the pedicle. The ligamentum flavum detachment of the target area is carefully performed not to injure the nerve root running below the distal end of the transverse process of the upper vertebrae. During ligamentum flavum detachment, the inferior edge of the cranial lamina and the superior margin of the caudal lamina are carefully removed for additional decompression and securing the operational field. At this time, care must be taken not to damage the facet joint. Thereafter, the nerve root is viewed and decompression is performed by checking the nerve running to the proximal part of the pedicle of the lower vertebrae for sufficient decompression along the nerve root to the distal end. After decompression is performed on the contralateral side in the same way, it is checked whether the bilateral running of the nerve root is without restriction. Then, hemostasis is performed at the surgical site, the surgical site is sutured and disinfected to complete the surgery.


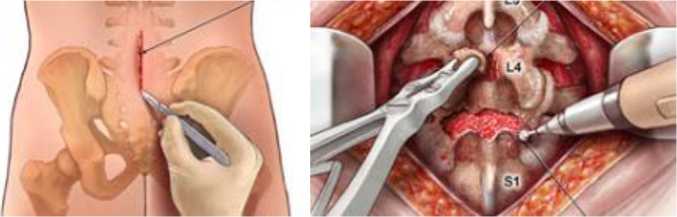


Setting the control group and randomization method

This study will be conducted by dividing the patients with lumbar spinal stenosis who will undergo posterior spinal decompression surgery into the interlaminar full-endoscopic laminectomy group (group 1) and conventional surgery group (group 2) through a prospective, randomized trial. Interlaminar full-endoscopic laminectomy is currently the preferred minimally invasive surgery method. Unilateral laminectomy bilateral decompression (ULBD) is a minimally invasive surgical method without endoscopy, and the clinical outcomes have been reported to show no significant difference from those of conventional surgical techniques. Therefore, it is necessary to verify the equivalence in clinical outcomes between the interlaminar full-endoscopic laminectomy and the conventional open decompressive laminectomy.

This is a prospective study, and among the subjects who voluntarily gave their consent to participate in the study and signed the ICF, those who are eligible according to the inclusion and exclusion criteria will be randomized to one of the two groups at a ratio of 1:1. The permuted block randomization method is used for randomization. Randomization is applied in sequence from subject number 1 using iCReaT, a web-based eCRF. The investigator will perform the surgery according to the results of randomization of the subjects. To minimize possible bias that may arise, randomization is performed by a researcher from the Department of Neurosurgery, and the surgeon is informed of the randomization code immediately before surgery.

This study has limitations in applying the double-blind method. It is inevitable that the investigator knows which procedure was used, and the patient also knows the surgical method due to the incision site after surgery. Therefore, the assessor-blind method, which applies the blinding only to the assessor, is applied, and an assessor is a third person who has not performed the surgery.

Statistical Consideration:

# Patients with lumbar spinal stenosis who will undergo uniportal endoscopic decompressive laminectomy

- Target sample size: 120 subjects (Study group: 60 subjects, Control group: 60 subjects, including 20 % drop-out rate)
- Primary outcome: ODI (Oswestry disability index)
- According to a previous study [1], the MCID (Minimal clinical important difference) of ODI was 12.8 and in another previous study [3], the standard deviation of the ODI value at 1 year after decompressive laminectomy was 18.8. Assuming an equivalence limit of 12.8, under the conditions of alpha = 0.05, power = 0.90, two-sided 95% confidence interval, and follow-up loss at 20%, 60 participants are needed in each group.


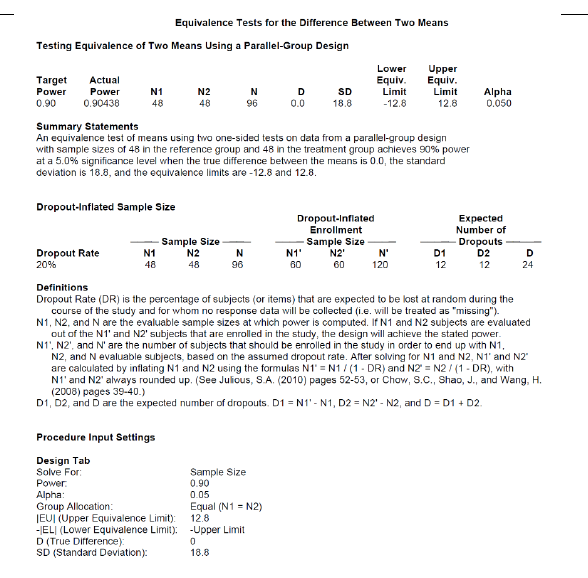


Subject Recruitment Plan

This is a prospective, multicenter study which will recruit subjects at the Department of Neurosurgery of Seoul St. Mary’s Hospital, Chundam Woorideul Spine Hospital and the Wiltse Memorial Hospital (Anyang). Those who are planning to undergo one-segment or two-segment decompression surgery for lumbar spinal stenosis will be recruited as subjects and advertisement for subject recruitment will not be used.

The surgical method investigated in this study is the most commonly used technique for lumbar spinal stenosis, and the clinical study is conducted through pre- and post-operative surveys and analysis of clinical and imaging data, so there is no harm to the subjects. However, if the subject refuses to participate in the study, the trial will not proceed with the subject. In addition, although this study is conducted on a patient group, there is no obligation or undue influence for participation, and there is no disadvantage to the patient for not participating in the study. Among those who voluntarily agreed to participate in this study and signed an informed consent form (ICF), those who met the inclusion criteria for this study and were not applicable to the exclusion criteria were selected, and subjects were randomly assigned to either of the study or control groups. The Principal Investigator (PI) of this study will not exclude subjects who are likely to participate in this study based solely on race or socioeconomic status. As long as the subject is eligible in terms of inclusion/exclusion criteria, the PI will make every effort to encourage the subjects to participate in the study, and inform the subjects of the objectives of this study so that they can properly represent the total of the patients who will undergo the treatment.

Assessment and Analysis of Study Results and Items for Observation

- **Demographic information:** Age, gender, and date of surgery of subjects

– **Physical examination and basic tests:** Medical history, smoking habits, basic blood tests, physical examination

– **Radiological tests:** The subjects undergo simple radiography and MRI (or CT) examination for baseline assessment. After surgery, during the hospitalization period, the degree of canal release, complications, and recurrence are examined through simple radiography and MRI (or CT) scans.

- **Oswestry disability index (ODI) questionnaire:** Scoring of the Oswestry disability index (10 items) is conducted at the visit. The investigator hands out the subject a questionnaire, and asks the subject to rate each item on a five-point scale for self-report. The rated score is expressed as a percentage of the total score of the answered questions excluding the questions not completed by the subject.

- **EQ5D-5L questionnaire:** The EQ5D-5L questionnaire (5 items) is conducted at the visit. The investigator hands out the subject a questionnaire and asks the subjects to rate and indicate each item for self-report. The total score of the indicated items is converted using the EQ5D value set for calculation.

- **VAS score ( pain scale):** The degree of pain felt by the subject during activity is assessed using a 100mm VAS (Visual Analogue Scale) for both lower back and the legs. The investigator explains the VAS to the subject and asks the subject to rate each item and indicate accordingly for self-report. In addition, the degree of pain in the surgical site (Postoperative Day 1 or Day 2) is also investigated using VAS.
- **Walking time:** The changes in values in the questions on walking ​​at postoperative 2, 12, 24, and 52 weeks are compared to the baseline values.
- **Satisfaction after surgery:** At the 1-year follow-up visit, the investigator hands out the questionnaire to the subject and asks the subjects to indicate for self-report.
- **Return to daily activities after surgery:** The time required to return to daily activities postoperatively (week)
- **Scar after surgery (POSAS[Patient and Observer Scar Assessment Scale] patient scale 2.0):** The patient assesses the postoperative scarring with 6 items and scored on a ten-point system, with a score of 1 indicative of normal skin and a score of 10 indicative of the worst scar imaginable, and the total score of the assessment is 60.

- **Other surgery-related outcomes:** The size of the surgical skin incision, postoperative drainage, operative time, duration of hospitalization (hours), postoperative Day 1 creatine kinase (CK, CPK) are also evaluated.

- **Presence of adverse events and complications:** Adverse events requiring revision surgery related to the intervention. When a patient arrives at the ward after completing the surgery, a resident (or fellow, clinical instructor, nurse, investigator, etc.) who is blinded to the surgical method examines the patient to determine whether there are any complications related to the intervention or any serious complications that require revision surgery.

Efficacy Assessment Criteria, Assessment Method, Interpretation

1. Primary Endpoint

The primary endpoint of this trial is the difference between the ODI score measured at baseline and that measured 12 months postoperatively.

Statistical analysis method: Equivalency will be declared if the upper and lower limits of the 95% confidence interval (CI) of the treatment difference value of each group’s ODI score at 12 months postoperatively fall within the pre-defined equivalence margin (+-12.8).

1. Secondary Endpoints

The secondary endpoints for this trial include clinical outcomes (VAS, EQ5D-5L, ODI, walking distance and time, satisfaction after surgery, time required to return to daily activities postoperatively, POSAS), radiographic outcomes, and other surgery-related outcomes.

Statistical analysis method: For each group, the differences from the baseline values to the postoperative values and differences in the pattern of change between the two groups are analyzed using repeated measures analysis of variance (ANOVA). In addition, for specific within-group or between-group comparisons at each time point, the t-test is used, while applying the adjusted significance level considering the multiplicity of the test.

Satisfaction after surgery, time required to return to daily activities postoperatively, Patient and Observer Scar Assessment Scale(POSAS scale)

Statistical analysis method: The satisfaction after surgery, the time to return to daily activities postoperatively, and POSAS scale score at the time of final postoperative follow-up are comparatively analyzed between the two groups using the t-test.

- Radiographic outcomes

Examination of the occurrence of complications during the final follow-up using simple radiographs (Development of spondylolisthesis, progression of degenerative lumbar spinal conditions, etc.), analysis of surgical outcomes through postoperative MRI (or CT)

- Statistical analysis method: The occurrence of complications is examined for each group, and Chi- square test or t-test is used for comparative analysis on the difference between the two groups for the degree of central canal release and the canal dimension measured using postoperative MRI or CT.

- Other surgery-related outcomes

Comparison between the two groups on other surgery-related outcomes. [size of the surgical skin incision, postoperative drainage, operative time, duration of hospitalization (hours), Postoperative (From immediately after surgery to discharge from the hospital), postoperative Day 1 creatine kinase (CK, CPK)]

Statistical analysis method: The degree of difference between the two groups is evaluated using the chi-square test or t-test.

General Statistical Analysis

General principles of analysis

- For continuous variables, descriptive statistics (number of subjects, mean, standard deviation, median, minimum, maximum) are presented, and for categorical variables, frequency (N) and percentage (%) are presented.

- In the analysis of the primary efficacy endpoint, if missing data occurs due to dropout, etc., the data are processed as a failure and the non-responder imputation (NRI) method is used.

- If the clinical trial is discontinued according to the discontinuation criteria, the data are handled as missing values.

- In the analysis of secondary efficacy endpoints, when there are missing values in the data, a method of last observation carried forward (LOCF) is used.

- For the analysis of the safety set, when there are missing values in the data, the data shall be analyzed as it is without performing imputation for the missing values (OC method).

Handling of analysis sets

- The analysis sets are categorized into safety set, full analysis set (FAS) and per protocol (PP) set.

- Efficacy data are analyzed using both FAS and PP, and final determination on efficacy endpoints is conducted by FAS analysis based on the modified ITT (intention to treat).

- Data on safety are analyzed for the safety sets.

- For the analysis of efficacy endpoints, when there are missing values in the data, the data shall be analyzed as it is without performing imputation for the missing values.

Definition of analysis sets

• FAS (Full Analysis Set): Subjects who met the inclusion/exclusion criteria and who underwent randomization to receive the intervention at least once during the trial period are included in FAS. In the efficacy analysis, regardless of the actual status of undergoing the surgery, analysis is performed based on the treatment group assigned by randomization.

• PP (Per Protocol Set): Subjects who have completed the trial without major protocol violations among the full analysis set (FAS) are included in PP set. However, if the trial is discontinued according to the discontinuation criteria, the clinical trial is considered to be completed and included in the PP analysis. For items of major protocol violations, see Section 13-5.

• Safety Set: Subjects who were randomized and have undergone the surgery at least once during the trial period are included in the safety set. In the safety analysis, analysis is performed based on the classification according to the actual type of surgery received.

|  | | | | | | |
| --- | --- | --- | --- | --- | --- | --- |
| **Patient Evaluation:**  **Schedule of Activities** | | | | | | |
| **Visit Type** | **Screening** | **Operation/**  **Treatment** | **Follow-up** | | | |
| **Visit** | **1** | **2** | **3** | **4** | **5** | **6** |
| **Visit week** | **-4~0weeks** | **0 day** | **2 weeks** | **12 weeks** | **24 weeks** | **52 weeks** |
|  |  |  | **± 5 days** | **± 4 weeks** | **± 8 weeks** | **± 8 weeks** |
| Subject written consent | ■ |  |  |  |  |  |
| Demographic information | ■ |  |  |  |  |  |
| Medical history/Surgical history^[1]^ | ■ |  |  |  |  |  |
| Physical examination | ■ |  |  |  |  |  |
| Clinical laboratory tests^[2]^ | ■ |  |  |  |  |  |

| Inclusion/Exclusion criteria | ■ | ■ |  |  |  |  |
| --- | --- | --- | --- | --- | --- | --- |
| Randomization |  | ■ |  |  |  |  |
| Surgery |  | ■ |  |  |  |  |
| MRI or CT | ■ | ■ |  |  |  |  |
| X-ray^[4]^ | ■ |  | ■ | ■ | ■ | ■ |
| ODI questionnaire | ■ |  | ■ | ■ | ■ | ■ |
| EQ-5D questionnaire | ■ |  | ■ | ■ | ■ | ■ |
| VAS questionnaire | ■ |  | ■ | ■ | ■ | ■ |
| Walking questionnaire |  |  | ■ | ■ | ■ | ■ |
| Other questionnaire^[5]^ |  |  | ■ | ■ | ■ | ■ |
| Collection of adverse events |  | ■ | ■ | ■ | ■ | ■ |
| * Visit 1 and visit 2 may proceed concurrently.   1. Investigation of medical history within 3 years from the time of screening (5 years in case of cancer) 2. Laboratory tests:   - Hematology (CBC): WBC, Hb, Hct, ESR - Biochemical profile (routine chemistry): hs-CRP, CPK   1. Simple radiography (X-ray): Simple radiographs will be obtained in the anteroposterior (AP), lateral, lateral-flexion, and lateral-extension views. In order to prevent undergoing excessive radiographic examinations, it is possible to replace the examination results with those taken within 4 weeks from the date of screening or follow-up visit. 2. Other questionnaires: satisfaction with the surgery, daily activities, POSAS scar questionnaire | | | | | | |

Expected Adverse Events/Risks and Countermeasures

The posterior spinal decompression surgery to be performed in this trial is a standard treatment for patients with lumbar spinal stenosis. There are no additional complications specifically from this study other than those related to general spinal surgery.

However, if AEs related to this clinical study occur, the investigator will take the following actions:

1. The investigator will immediately report to the IRB upon discovering the adverse event.
2. The investigator will take necessary medical action.

If the subject requires emergency measures due to this study, the necessary emergency measures shall be taken as soon as possible. In addition, in the case of serious adverse events (SAEs), prompt and appropriate actions shall be taken to minimize possible damage. However, general complications expected after lumbar spinal surgery are not considered as complications “caused by this study.”

* Discontinuation•Drop-out Criteria

The principal investigator may consider discontinuation of the clinical study in the following cases.

1. In the event of serious adverse events (SAEs) in subjects
2. Other cases where the investigator deems that continuation of the study may be difficult

The cases for consideration of subject dropout are as follows.

1. Non-compliance to the instructions of the investigator
2. If the subject or LAR withdraws the consent to participate (presented explicit wishes to refuse the participation in the study)
3. Violation of the inclusion/exclusion criteria
4. In the event of serious adverse events SAEs/adverse device effect (ADE)
5. Other cases where the investigator deems that continuation of the study may be difficult

In the event of a withdrawal or dropout of a subject, the investigator must make every effort to follow-up the study subject who has withdrawn or dropped out for any reason, and must perform a follow-up monitoring evaluation for safety.

**Assessment Criteria, Assessment Method and reporting of Safety, including Adverse Events**

Adverse events occurring during the clinical trial are followed up until the symptoms are resolved or stabilized. Adverse events that occur are coded using Preferred Terms (PTs) according to the Medical Dictionary for regulatory activities (MED-DRA). All adverse events (AEs) and serious adverse events (SAEs) that occurred after treatment and the unexpected adverse events (UAEs) related to the treatment in the trial are summarized according to the severity by the time point of onset using Preferred Terms (PTs) and prepared in graphs. These are recorded based on the self-report of the subjects and examination during their visits. In recording the AE-related information, the name and duration, the scope and severity of symptoms, the causal relation with the medical device, additional treatment, the outcome of AEs, and the status of seriousness are described in detail in the Special Form of the case report form (CRF).

Statistical analysis method: In this study, AEs indicate cases of new or worsened symptoms or signs that were not observed during the baseline assessment and include all signs, symptoms and diseases regardless of the causal relation with the medical device. In the event of AEs, the name of the related symptoms, time of onset, duration, severity of symptoms, and causal relation with the investigational device are recorded in CRF. The number of cases and the number of applicable subjects are calculated for each case of AEs, and the rate of occurrence of adverse events 95% two-sided confidence intervals are presented. The difference in the rate of AE occurrence between the two groups is compared using the chi-square test or Fisher's exact test.

Protocol Abstract(cont'd)

Estimated Accrual:

Target sample size 120 subjects (Study group 60 subjects, control group 60 subjects, including 20% drop-out rate)

Phase:

□ Phase I □ Phase IIa □ Phase IIb □ Phase III □ Phase IV □ Phase I/II ■ Other

Site of Study:

This protocol is performed as an:

■Inpatient ■Outpatient □Community □Ect. ( )

Length of Stay : (What is the length and frequency of hospitalization)

Follow-up will be performed on the day of surgery as well as 2 weeks, 3 months, 1 months and 1 year postoperatively.

Where will study be conducted:

□ Only at WMH □ Community ■ Multicenter ( ■Domestic □ Multinational)

Name of Sponsor / Funding Source:

Not applicable

Sponsor Contact / Company Address / Telephone / Fax:

Not applicable

Competing Protocol: (Protocol No. & Date)

Name of Research Coordinator / Pharmacist (name /position) / Data Manager Responsible for Protocol

Please do not delete the items in the form and mark non-applicable items as “Not applicable” or “N/A”.
