# Supplementary material for: Evaluation of the efficacy and safety of conventional and interlaminar full-endoscopic decompressive laminectomy to treat lumbar spinal stenosis (ENDO-F trial): Protocol for a prospective, randomized, multicenter trial
Source: PLoS One. 2023 Apr 5;18(4):e0283924. doi: 10.1371/journal.pone.0283924 (PMC10075401; doi:10.1371/journal.pone.0283924)
Supplement: S6 File — (DOCX) [file pone.0283924.s006.docx]

|  | Wooridul Hospital Clinical Study Protocol  Date Ver 1.2021.04.15  Ver 1-1.2021.07.27 |
| --- | --- |
|  | Basic Information |
| **1. Study Title** | English: Evaluation of the efficacy and safety of conventional and interlaminar full-endoscopic decompressive laminectomy to treat lumbar spinal stenosis (ENDO-F Trial): A prospective, randomized, Assessor blind, multicenter trial |
| **2. Stage** | □ Validity (Pilot Study) □ Clinical Trials for Product Approval □ Post-Marketing Research ■ Academic Research |
| **3. Institution & Address** | Wooridul Hospital, 47-4 Cheongdam-dong, Gangnam-gu, Seoul |
| **4. Sponsor** | □ Wooridul Spine Foundation □ Other – Name of Corporate:  Name of CEO:  Name of Person In Charge  Address: |
| **5. Investigators** | **Principal investigator**  Name Affliliation Completion of Training Name/Date of Training  Junseok Bae Neurosurgery Y CELP_GCP(Good Clinical Practice Training)/2021.04.05.  **Subinvestigators (Co-investigators)**  Name Affliliation Completion of Training Name/Date of Training  Sang-ha Shin Neurosurgery Y CELP_GCP(Good Clinical Practice Training)/2021.04.14.  Sang Soo Eun Neurosurgery Y CELP_GCP(Good Clinical Practice Training)/2021.04.15.  Han Joong Keum Neurosurgery Y CELP_GCP(Good Clinical Practice Training)/2021.01.27.  Young Soo Choi Neurosurgery Y CELP_GCP(Good Clinical Practice Training)/2020.08.21.  Seong Gyun Jeong, Fellow Y CELP_GCP(Good Clinical Practice Training)/2021.07.26  **CRC**  Name Affliliation Completion of Training Name/Date of Training  Yoon Joo Lee Academic Promotions Team Y CELP_GCP(Good Clinical Practice Training)/2021.02.18 |
|  | **6. Configuration**  Data Acquirement  Recruitment of Patients and Acquirement of Data: Junseok Bae, Sangha Shin, Sangsu Eun, Hanjoong Geum, Yongsoo Choi |
| Participating Institutes | \| Institute Name \| Address \| \| --- \| --- \| \| Seoul St Mary’s Hospital,  The Catholic University of Korea \| Seoul St Mary’s Hospital of The Catholic University of Korea, 222, Banpo-daero, Banpo-dong, Seocho-gu, Seoul \| \| Wooridul Hospital \| 47-4, Cheongdam-dong, Gangnam-gu, Seoul \| \| Wiltse Memorial Hospital (Anyang) \| 560, Gyeongsu-daero, Dongan-gu, Anyang-si (Hogye-dong) \| |
| Statistical Analysis | Principal Investigator |
| Administration, Technology or Data Support | Yoon Joo Lee |
| Manager of Devices for Clinical Use | N/A |

**7.** Study Objectives

7-1) Main Objective: The utilization of endoscopic surgery in lumbar spinal stenosis was investigated to compare the clinical results with conventional palliative surgery to confirm whether identical results are produced. In addition, the prospective clinical data of endoscopic surgery are analyzed to determine the basis for clinical practice guidelines, and the clinical practice guidelines are proposed to assist in selecting the appropriate treatment. Therefore, a total of three institutes participated in the comparison study of single-hole endoscopic posterior decompression in patients with lumbar spinal stenosis with the conventional palliative surgery methods.

7-2) Secondary Objectives N/A

1. **Study Background and Significance (Referenced Lieratures Attached)**

According to the Health Insurance Review and Assessment Service (HIRA), the number of patients with spinal diseases, the target population of this study, was reported to be around 3.63 million in 2018 alone, showing an increase of 450,000 patients over the past five years. In particular, the number of patients with lumbar spinal stenosis (LSS) increased by approximately 32.4% in 5 years. According to the 2018 Statistical Yearbook of Surgery by the National Health Insurance, there were 7,218 cases of endoscopic spine surgery, an increase of 41.3% from 5,108 cases in the previous year, and the treatment cost also increased by 45.9%. Compared to the increase of 2.4% in the number of conventional spine surgeries from 165,573 cases to 169,706 cases for the same period, a significant increase is confirmed, indicating that the cases of endoscopic spine surgery show a trend of remarkable growth. In addition, the prevalence of spinal diseases is expected to gradually escalate with the increase in the senior population, and the burden of increased medical expenses for the treatment is also projected.

Posterior decompression in lumbar spinal stenosis and posterior lumbar discectomy in lumbar disc herniation are the most conventional methods used to resolve the patient's symptoms. The conventional methods have problems in terms of much bleeding, postoperative pain, instability and a decrease of muscles around the spine, and therefore, minimally invasive surgery is performed to preserve anatomical structures. Unilateral laminectomy bilateral decompression (ULBD) is the representative, most commonly used minimally invasive technique, and other methods such as spinous process osteotomy and endoscopy are also used. It has been reported that minimally invasive surgery has a number of advantages compared to the conventional surgical technique, and the clinical outcomes are not different from those of the conventional open laminectomy.

Recently, **surgical techniques of spinal decompression and discectomy using endoscopy** have been developed and applied in clinical practice. The endoscopic spine surgery is classified into uniportal endoscopic technique and biportal endoscopic technique depending on the number of ports for insertion of instruments. Since the surgical site is accessed through a skin incision of less than 1 cm, damage to the normal structure can be minimized, resulting in fewer postoperative complications such as pain and epidural adhesion.
However, although interlaminar full-endoscopic laminectomy has been reported with **good clinical outcomes in retrospective studies, the clinical outcomes have not been confirmed in a multicenter, prospective, randomized clinical trial**. In addition, while the conventional open laminectomy and microscopic surgery currently performed on patients with lumbar spinal stenosis are covered by national health insurance and are appropriately applied for patients in need of surgical treatments, endoscopic spine surgery are not fully accepted for health insurance coverage, and thus interlaminar full-endoscopic laminectomy has been performed only for some of the patients with lumbar disc herniation. Most of the interlaminar full-endoscopic laminectomy studies reported in Korea are retrospective studies, and there has been no level 1 study to establish the efficacy and safety of the endoscopic surgery compared to the conventional open laminectomy. Therefore, in this study, the efficacy and safety of interlaminar full-endoscopic laminectomy were compared with those of conventional open laminectomy with established clinical outcomes through a multicenter, prospective randomized trial.

1. **Type of Study**
    _
2. ■ Prospective □ Retrospective
3. Experimental Study: ■ Randomized Control Study □ Non-randomized Study

Observational Study: □ Cohort Study □ Case-Control Study □ Cross-Sectional Study (□ Prevalence Study, □ Ecological Study，□ Sensitivity and Specificity Study)

**10. Hypothesis**

This study aims to verify the equivalence of clinical outcomes of the endoscopic surgical method in lumbar spinals tenosis to those of a conventional surgical technique.

1. Is it necessary to obtain clinical trial approval from the Director of the Ministry of Food and Drug Safety for the clinical trial to be used for purposes other than the licensing of medical devices or pharmaceuticals?

[Investigational Device Exemption(IDE)]

□ Yes (Studies that require clinical trial approval from the Director of the Ministry of Food and Drug Safety)
 ■ Not Applicable

**Study Design**

1. **Explanation of Interventional Procedures and Devices**

1) Interlaminar Full-Endoscopic Decompressive Laminectomy

As shown in the figure below, decompressive laminectomy is performed using endoscopic instruments and instruments for spinal surgery. The method can minimize the injury to normal tissue. After making a skin incision of about 1 cm, the endoscope is introduced, the location is checked on the X-ray, and high frequency is applied to control bleeding. After exposing the lamina, a drill for endoscopy is used to perform laminectomy while minimizing the injury of the facet joint to expose the ligamentum flavum. During the process of ligamentum flavum detachment, the inferior edge of cranial lamina and superior margin of the caudal lamina are carefully removed if necessary for additional decompression and to secure operation field for improved visualization.


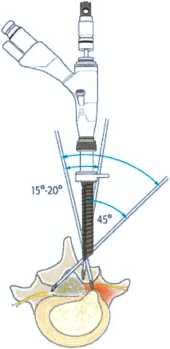

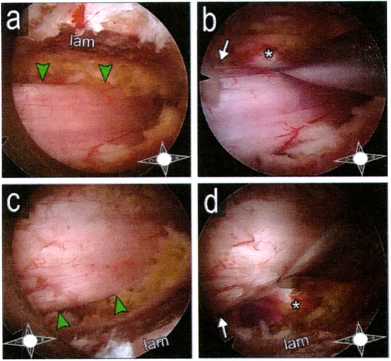


**2)** Conventional Open Decompressive Laminectomy

This method is the commonly applied technique of laminectomy in which a midline incision is made and the bilateral hypertrophied ligamentum flavum is removed with open bilateral access. After disinfection, the surgical site is located with a simple radiograph of the lateral view for the lumbar spine. A skin incision of about 3 cm in length is made from the midline of the surgical site to the long axis, and this is sufficient to secure the operational field. After detaching the paraspinal muscles from the spinous process, lamina, and vertebral joint, the fascial flap is opened and the operational field is secured using a retractor.

In the intervertebral foramen, the proximal ligamentum flavum is clearly visible in the transverse process of the upper vertebrae and the distal face of the pedicle. The ligamentum flavum detachment of the target area is carefully performed not to injure the nerve root running below the distal end of the transverse process of the upper vertebrae. During ligamentum flavum detachment, the inferior edge of the cranial lamina and the superior margin of the caudal lamina are carefully removed for additional decompression and securing the operational field. At this time, care must be taken not to damage the facet joint. Thereafter, the nerve root is checked and decompression is performed by checking the nerve running to the proximal part of the pedicle of the lower vertebrae to ensure sufficient decompression along the nerve root to the distal end. After decompression is performed on the contralateral side in the same way, it is checked whether the bilateral running of the nerve root occurs without restriction. Then, hemostasis is performed at the surgical site, the surgical site is sutured and disinfected to complete the surgery.


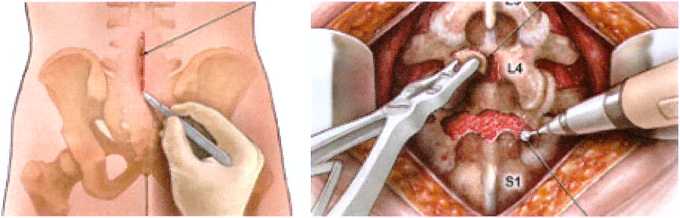


| **13.** Application | ■ Not Applicable | | | |
| --- | --- | --- | --- | --- |
| Device Name | Corporate Name | Location/Address | Approval of KFDA | Solution if device becomes unusable |
|  | □ Yes □ No | | | |
|  | □ Yes □ No | | | |

**14.** Study Period

| Date of IRB Approval ~ 2024.12.31 | | |
| --- | --- | --- |
| Category | Expected Period | Period |
| Recruitment | Months | Date of IRB Approval~2024.12.31 |

| Clinical Investigation |  | Months | Date of IRB Approval ~2024.12.31 |
| --- | --- | --- | --- |
| Data Organization | [1 | 2 Months | 2024.01.01 ~2024.02.28 |
| Statistical Work |  | 2 Months | 2024.03.01 ~2024.04.30 |
| Paper Write-Up |  | 8 Months | 2024.05.01-2024.12.31 |
| Total |  | Approximately 3 years, 8 months | Date of IRB Approval ~2024.12.31 |
| **15.** | Study Subjects | | |

Patients with ≥ Grade B lumbar central canal stenosis who agree to undergo one to two segment posterior spinal decompression surgery, and are willing to participate and comply with the 1-year follow-up protocol.

Criteria

Prospective Expected Period of Treatment including Medical Procedures and Surgery

Retrospective Period of Treatment including Medical Procedures and Surgery

Sample Size and Calculation Background

Date of IRB Approval-2024.12.31

■ Not Applicable

# Patients with lumbar spinal stenosis who will undergo uniportal endoscopic decompressive laminectomy

- Target Sample Size: n=120 (Study group: 60, Control group: 60, including dropout rate at 20%)
- Primary outcome: ODI (Oswestry disability index)
- - According to a report of an existing research [1], the MCID (minimal clinical important difference) of ODI was 12.8, and in another previous study [3], the standard deviation of the ODI value at 1 year after decompressive laminectomy was 18.8. Assuming an

equivalence limit of 12.8, under the conditions of alpha = 0.05, power = 0.90, two-sided 95% confidence interval, and follow-up loss at 20%, 60 participants are needed for each group, and 40 participants are to be recruited from Wooridul Hospital.


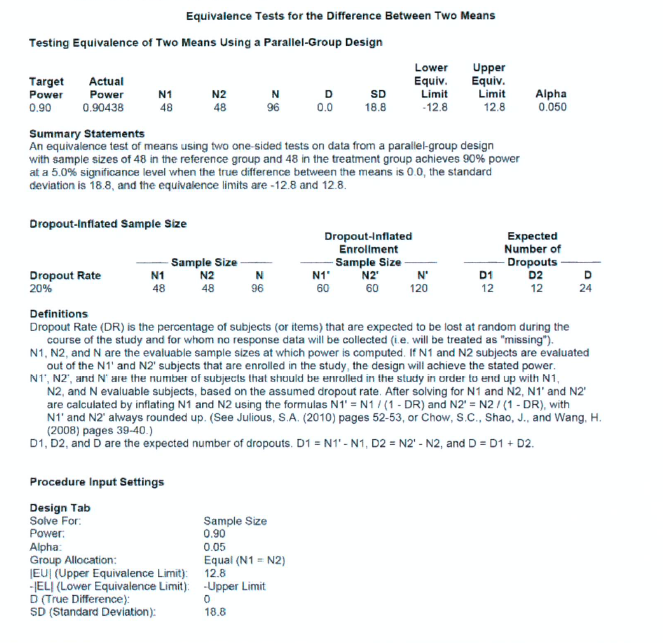


| Inclusion Criteria | 1. Patients with ages between 20~80 years 2. Patients ≥ Grade B lumbar central canal stenosis who agree to undergo one or two segment posterior spinal decompression surgery 3. Patients who are willing to participate and fully comply with the 1-year follow-up protocol 4. Participants that have signed an agreement form after he or she (if signature is available) or a legally authorized representative fully understood the contents of the clinical trial. |
| --- | --- |
| Exclusion Criteria | 1. Patients with spondylolisthesis of more than Meyer Gr II 2. Patients who have a history of lumbar spinal surgery 3. Patients with degenerative lumbar scoliosis (Cobb angle > 20^o^) 4. Patients with lumbar spinal stenosis that is not degenerative by nature or is caused by lumar disc herniation. 5. Patients with other spinal disorders in the lesions of the lumbar spinal stenosis (e.g ankylosing spondylitis, spinal tumor, spinal fracture etc.) 6. Patients with psychological disorders (e.g dementia, mental retardation, or drug addiction) 7. Patients who refused to participate in the study 8. Patients who were deemed unfit for the trial by their physicians |
| Control Group | Conventional Open Decompressive Laminectomy |
| Recruitment Method | Outpatients and Inpatients |
| Patient Allocation (Randomization etc.) | - This is a prospective, multicenter, assessor-blind, randomized, controlled trial. - According to the patient group assigned to each sub-task, the trial is conducted by dividing the patients into an interlaminar full-endoscopic decompressive laminectomy group (group 1) and a conventional open decompressive laminectomy group (group 2). - Apart from the difference in the surgical technique, the two groups undergo the same treatment and follow-up (day of surgery, postoperative 2 weeks, 3 months, 6 months, 1 year) preoperatively and postoperatively, and variables for radiographic and clinical outcomes of the surgery are recorded to compare the differences between the two groups. Patients scheduled to be enrolled in this study are briefed on the objectives and methods of the clinical trial from the investigators prior to the study participation and are enrolled in the study by signing the agreement form. The screening assignment number consists of a series of randomized six-digit numbers. The first digit represents the study institution, the second digit indicates the sub-task, the third digit is S for Screening, and the remaining three digits indicate the serial number for enrollment (Example: B1S-001 -> First Patient Enrolled for Screening for Surgery 1 at Seoul National University Bundang Hospital). - Among the participants who voluntarily gave their consent to participate in the study and signed the agreement form, those who are eligible according to the inclusion and exclusion criteria are randomly assigned to one of the two groups at a ratio of 1:1 - The permuted block randomization method was used for randomization. Randomization is applied in sequence from participant number 1 using iCReaT, a web-based eCRF. The investigator will perform the surgery according to the results of the randomization of the subjects. To minimize possible bias that may arise, randomization is performed by a researcher, and the surgeon is informed of the randomization code immediately before surgery.   This study has limitations in applying the double-blind method. It is inevitable that the investigator will find out which procedure was used, and the patient will also know the surgical method used on them due to the incision site after surgery. Therefore, the assessor-blind method, which applies the blinding only to the assessor, is utilized, and the assessor is designated ot be a third person who has not performed the surgery. |
|  |  |

- The randomized number consists of a series of five-digit numbers. The first digit of the randomization number represents the study institution, the second digit indicates the sub-task, and the remaining three digits indicate the serial number for enrollment (Example: B1-001 -> First Enrolled Patient for Surgery 1 at Seoul National University Bundang Hospital).
- All basic examinations and surveys will be conducted for evaluation before surgery, on the day of surgery, and at follow-up at 2 weeks, 3 months, 6 months, and 1 year postoperatively. Preoperative radiographic examination, clinically physical examination (age, gender), medical history taking (past medical history, surgical history) are performed on the study participants and results of the Lab tests (CBC, Routine chemistry) are checked before the surgery. At baseline and during the preoperative hospitalization period (X-ray, MRI or CT), postoperative 2 weeks, 3 months, 6 months, and 12 months, radiographic examinations (X-ray) are performed to examine the status of occurrence of complications. At baseline, immediately after the surgery (X-ray, MRI or CT), postoperative 2 weeks, 3 months, 6 months, and 12 months, the VAS, ODI, and EQ5D, walking distance, satisfaction, and POSAS are surveyed to evaluate clinical outcomes. Immediately after the surgery, other surgery-related outcomes (postoperative bleeding, operative time, period of hospitalization, postoperative day 1 creatine kinase (CK, CPK)) are measured for comparison of the surgery-related items.

Research Method

- However, if the subject is unable to pay a visit at the scheduled time due to unavoidable reasons (e.g., safety issues due to the COVID-19 pandemic), non-face-to-face meeting or telephone surveys allowed by the current law may be applied instead of the actual outpatient visit.

**16. Measurement of the Results**

VAS, ODI, EQ5D

Clinical Physical and Neurological Test Results

Blood Test

Radiograph of the Lumbar and the Whole Spine

Radiological Computerized Tomography Scan of the Lumbar

Magnetic Resonance Imaging of the Lumbar

Other Satisfaction of the Surgery, POSAS Wound Survey

**17. Data Management**

VAS, EQ5D-5L, ODI, walking distance and time

Postoperative changes in the scores of the clinical outcomes against the preoperative scores at each time point

Statistical Analysis Method : For each group, the differences from the preoperative values to the postoperative values and differences in the pattern of change between the two groups are analyzed using repeated measures analysis of variance (ANOVA). In addition, t-tests are used for specific comparisons within or between groups, comparisons at each time point, while applying the adjusted significance level due to the multiplicity of the test.

Satisfaction after surgery, time required to return to daily activities postoperatively, Patient and Observer Scar Assessment Scale(POSAS scale)

Statistical Analysis Method: The satisfaction after surgery, the time to return to daily activities postoperatively, and POSAS scale score at the time of final postoperative follow-up are comparatively analyzed between the two groups using the t-test.

Radiographic Outcomes

Examination of the occurrence of complications during the final follow-up using simple radiographs (Development of spondylolisthesis, progression of degenerative lumbar spinal conditions, etc.), analysis of surgical outcomes through postoperative MRI (or CT)

Statistical Analysis Method: The occurrence of complications is examined

Statistical Analysis Method

for each group, and a Chi- square test or a t-test is used for comparative analysis on the difference between the two groups regarding the degree of disk disectomy measured using postoperative MRI or CT.

Other surgery-related outcomes

Comparison between the two groups on other surgery-related outcomes. [size of the surgical incision, postoperative bleeding, operative time, duration of hospitalization (hours), Postoperative (From immediately after surgery to discharge from the hospital), postoperative Day 1 creatine kinase (CK, CPK)]

Statistical Analysis Method: The degree of difference between the two groups is evaluated using the chi-square test or t-test.

General Principle of Analysis:

. -For continuous variables, descriptive statistics (number of participants, mean, standard deviation, median, minimum value, maximum value) are presented, and, frequency (N) and percentage (%) are presented for categorical variables.

Handling of Analysis Sets

-The analysis sets are categorized into safety sets, full analysis sets (FAS) and per protocol (PP) sets.

■ Efficacy data are analyzed using both FAS and PP, and the final determination on the efficacy evaluation parameters is conducted by FAS analysis, whch is the analysis based on the modified ITT (intention to treat)

■ Data on safety are analyzed for the safety sets.

• When there are missing values in the data regarding efficacy evaulation, the data shall be analyzed as it is without performing imputation for the missing values.

Definition of Analysis Sets

•FAS (Full Analysis Set) Subjects: Participants who met the inclusion/exclusion criteria and who underwent randomization allocation to receive the surgery at least once during the trial period are included in FAS. In the efficacy analysis, analysis is performed based on the treatment group assigned by randomization regardless of the actual status of undergoing the surgery.

•PP (Per Protocol Set) Subjects: Participants who have completed the trial without major protocol violations among the full analysis set (FAS) are included in the PP set. However, if the trial is discontinued according to the discontinuation criteria, the clinical trial is considered to be completed and included in the PP analysis. See Section 13.5 for the article on major protocol violations.

• Safety Set Subjects: Participants who were randomized and have undergone the surgery at least once during the trial period are included in the safety set. In the safety analysis, analysis is performed based on the actual type of surgery the participant received.

Case Report Format Case Report (Collecting Patient Information): Attached

■ If missing data occurs due to dropouts, etc., in the analysis of the primary efficacy endpoint, the data is processed as a failure and the non-responder impuatation (NRI) method is used.

**1)** Missing Values ■ If the clinical trial is discontinued according to the discontinuation criteria, the data is processed as missing values

+ If missing data occurs in the analysis of secondary efficacy endpoints, the last observation carried forward (LOCF) method is used.

•If missing data occurs in the analysis of safety sets, the data is analyzed as it is without performing imputation for the missing values (OC method).

18. Hypothesis

This study will prove to be a valuable asset by confirming that the efficacy and stability of endoscopic surgery portrays no differences to the clinical outcomes of the conventional open decompression, for which the clinical outcomes are already well-established.

Ethical Issues

**19.** Prior Consent (Both Patient and Doctor)

■ Required □ Not Required

**20.** Estimated Costs (Estimated Costs of Research)

| Category | Details | Amount | Total Estimated Cost |  |
| --- | --- | --- | --- | --- |
| Printing Costs | N/A | N/A | N/A |  |
| Charge for Information DB Usage | N/A | N/A | N/A |  |
| Material Costs (Research Materials etc.) | N/A | N/A | N/A |  |
| Computer Processing Costs | N/A | N/A | N/A |  |
| Utility Costs (Phone/Electricity etc) | N/A | N/A | N/A |  |
| IRB Review Fee | Initial Review Costs | 500,000 | 500,000 |  |
| Indirect Expenses | N/A | N/A | N/A |  |
| Total | N/A | N/A | 500,000 |  |

**21. Benefit for the Patient upon Participation**

- N/A

1. Public Statement on the Conflict of Interest

■ I declare that I have no financial or conflicting relationships with the topics, data, and organizations mentioned in this paper.

□ I declare that I have at least one conflicting interest.

Conflict of Interest:

、Example: Employment, Affiliation to Consultants，Possession of Capital Sock, Commission

( )

1. Alternative Treatments

| - | ■ Not Applicable |
| --- | --- |
| **24.** Potential Danger (Write ratio if required) | |
| - | ■ Not Applicable |
| **25.** Potential Benefits (Write ratio if required) | |
| - | ■ Not Applicable |
| **26. Report on Adverse Effects and Precautions for Use** | |

* Anticipated Adverse Effects/Risks and Countermeasures

Lumbar open compression is a standard treatment generally utilized to treat lumbar spinal stenosis. There are no additional complications resulting from this study other than those that can occur in general spinal surgery (bleeding, infection, nerve damage etc.).

However, if adverse effects related to this study do occur, the principal investigator must conduct the following measures.

1. If a doctor discovers any side-effects, he/she must report to the IRB immediately.

® The doctor takes any necessary medical measures.

Emergency measures need to be conducted as soon as possible if they need to be conducted due to damage being inflicted on the research participants. In addition, immediate and appropriate measures must be taken to minimize potential damage in the event of a serious adverse event.

However, complications that are expected to occur after a typical lumbar surgery is not considered as complications “caused by this study”.

**27. Evaluation and Reporting of Safety including Adverse Events**

Adverse events occurring during the clinical trial are followed up until the symptoms are resolved or stabilized. Adverse events that occur are coded using preferred terms according to the Medical Dictionary for Regulatory Activities (MED-DRA). All adverse events (AEs) and serious adverse events (SAEs) that occurred after the treatment and the unexpected adverse events (UAEs) related to the treatment in the trial are summarized according to the severity by the time point of onset using preferred terms and are illustrated as graphs. These are recorded based on the self-report of the participants and examination during their visits. The name and duration, the scope and severity of symptoms, the causal relation with the medical device, additional treatment, the outcome of AEs, and the severity are described in detail in the Special Form of the case report form (CRF).

Statistical Analysis Method: In this study, adverse effects indicate cases of new or worsened symptoms or those that were not observed during the surgery and include all signs, symptoms and diseases regardless of the causal relation with the medical device. In the event of adverse effects, the name of the related symptoms, time of onset, duration, severity of symptoms, and causal relation with the investigational device are recorded in the CRF. The number of cases and the number of applicable participants are calculated for each case of adverse effects that occurred, and the rate of occurrence of adverse events and 95% two-sided confidence intervals are presented. The difference in the rate of occurrence of adverse effects between the two groups is compared using the chi-square test or Fisher's exact test.

**28.** Cancer-Related Research

- ■ Not Applicable

1. Protocol on Patient Damage Compensation

-'Protocol on Damage Compensation for Research Participants’ (Attached), Insurance Policies (Attached)

1. Confidentiality of Clinical Records and Information of Patients

Confidentiality of all the information regarding the research participants must be ensured. For all study-related records, the patient’s EMR number and hospital registration number must be kept in separate files under the responsibility of the principal investigator, and they shall be coded to prevent the identification of personal information while accessing the study data. Alternatively, the study data can be saved in a password-protected file and stored in a locked laboratory. Each study participant is assigned an identification code number to prevent the identification of personal information, and their names are recorded with initials. The principal investigator must ensure the confidentiality of all information related to this study. According to Article 15 of the Enforcement Rules of the Bioethics and Safety Act, the study-related records must be kept for 3 years from the period of study termination, and when the data retention period is over, the data must be destroyed according to Article 16 of the Enforcement Decree of the Personal Information Protection Act. However, if the plan for retention of records is changed for follow-up trials, records, data accumulation, etc., the records can be retained for a longer period. The information of this study can only be disclosed to IRB or related investigators. The principal investigator may only use the results of this study for the purposes of registration, publication, and provision of information to medical and pharmaceutical experts.

- I hereby declare to keep the information regarding patients confidential.
  Signature *Junseok Bae*

|  |  |
| --- | --- |
| Administrative Work |  |
| **31.** Supporting Departments | |
| - Academic Promotions | Team |
| **32.** Location of Data and Tools | |
| **- N/A** |  |
| **33.** | Notice of Outcome to Patient |
| - | ■ Not Applicable |
|  |  |
| Principal Investigator | Signature Date |

- This study does not require approval from IRB

Approval of the Institutional Review Board □ Approved (Date: ) □ Rejected

A plan must be submitted to the Ministry of Food and Drug Safety for uses other than the licensing of medical devices or pharmaceutical products of the Ministry of Food and Drug Safety.

- Not Required. Clinical Trials that fall into the category of licensing of medical devices or pharmaceuticals
- Approval received from the Minister of Food and Drug Safety for clinical trials other than the licensing of medical devices or pharmaceuticals
- Approval not received from the Minister of Food and Drug Safety for clinical trials other than the licensing of medical devices or pharmaceuticals

Head of Hospital (Head of Hospital) Signature Date

Chairman of the Board Signature

Date

**APPENDIX**

1. Reference (Abstracts of Related Literature): Attached
2. Case Report Form (Case report Form: Organization of Patient Data): Attached
3. Informed Consent (Agreement for Study Cooperation: Patient, Other GPs)
4. PI (Principal Investigator) Profile
5. Reference (Abstracts of Related Literature)
6. Wing P, Cheung H, King C, Wong H, La니 ST, Cost analysis comparison between conventional microsurgical decompression and full-endoscopic interlaminar decompression for lumbar spinal stenosis surgery. J Spine Surg. 2020 Dec;6(4):721-728. doi: 10.21037/jss-20-552.
7. Chen KT, Choi KC, Song MS, Jabri H, Lokanath YK, Kim JS. Hybrid Interlaminar Endoscopic Lumbar Decompression in Disc Herniation Combined With Spinal Stenosis. Oper Neurosurg (Hagerstown). 2021 Feb 16;20(3):E168-E174. doi: 10.1093/ons/opaa360.
