# Supplementary material for: Evaluation of the efficacy and safety of conventional and interlaminar full-endoscopic decompressive laminectomy to treat lumbar spinal stenosis (ENDO-F trial): Protocol for a prospective, randomized, multicenter trial
Source: PLoS One. 2023 Apr 5;18(4):e0283924. doi: 10.1371/journal.pone.0283924 (PMC10075401; doi:10.1371/journal.pone.0283924)
Supplement: S7 File — (PDF) [file pone.0283924.s007.pdf]

우리들병원 임상 연구 계획서

날짜 Ver 1. 2021.04.15

Ver 1-1. 2021.07.27

기본 자료

1. 연구 제목	국문: 요추부 협착증에서 후궁간 경유 경피적 단일공 내시경하 후방감압술과 고식적 후방 감압술의 임상적 및 방사선학적 유효성 및 안전성 평가 (ENDO-F Trial): 전향적, 무작위 배정, 평가자 눈가림, 다기관 연구<br>영문: Evaluation of the efficacy and safety of conventional and interlaminar full-endoscopic decompressive laminectomy to treat lumbar spinal stenosis (ENDO-F Trial): A prospective, randomized, Assessor blind, multicenter trial
2. 단계	□ 타당성(시범연구) □ 품목 허가용 임상시험 □ 시판 후 연구 ■ 학술연구
3. 기관 & 주소	서울시 강남구 청담동 47-4, 우리들 병원
4. 지원	□ 우리들척추재단 □ 기타 - 회사명:<br>대표 명: 담당자 명:<br>주소:

5. 연구자

	이름	소속	교육이수 완료	교육명/날짜
책임연구자	배준석	신경외과	Y	CELP_GCP(Good Clinical Practice Training)/ 2021.04.05
시험담당자 (공동연구자)	신상하	신경외과	Y	CELP_GCP(Good Clinical Practice Training)/ 2021.04.14
	은상수	신경외과	Y	CELP_GCP(Good Clinical Practice Training)/ 2020.04.15
	금한중	신경외과	Y	CELP_GCP(Good Clinical Practice Training)/ 2021.01.27
	최용수	신경외과	Y	CELP_GCP(Good Clinical Practice Training)/ 2020.08.21
	정성균	전임전문의	Y	CELP_GCP(Good Clinical Practice Training)/2021.07.26
CRC	이윤주	학술홍보팀	Y	CELP_GCP(Good Clinical Practice Training)/ 2021.02.18

6. 구성

자료 획득: 환자 모집 및 자료 획득: 배준석, 신상하, 은상수, 금한중, 최용수

참여기관명:

기관명	주소
가톨릭대학교 서울성모병원	서울시 서초구 반포동 반포대로 222, 서울성모 병원 신경외과
우리들병원	서울시 강남구 청담동 47-4
윌스기념병원 (안양)	안양시 동안구 경수대로 560(호계동)

통계분석	연구책임자
행정, 기술, 혹은 자료 지원	이윤주
임상시험용 기기 관리자	성명 N/A 소속 N/A

7. 목적

7-1) 주요목적 척추관 협착증에서 내시경 수술 방법이 기존 고식적 수술과 비교하여 **임상적 결과의 동등성을 확인해 보고**

자 한다. 또한 내시경 수술의 전향적 임상데이터를 분석하여 **임상 진료 지침에 대한 근거를** 마련하고, 치료법 선택에 대한 임상 진료 지침을 제시하고자 한다. 이를 위해 총 3개 기관에서 요추 협착증 환자에서 단일공 내시경을 이용한 후방 감압술의 **임상적 효용성 및 안정성을** 기존의 고식적 수술법과 비교해 보고자 한다

7-2) 이차목적 N/A

8. 연구계획의 배경 및 유의성(참고문헌 첨부)

본 연구에서 대상으로 하는 척추 질환 환자는 건강심사평가원에 따르면 2018년 한 해에만 약 363만명으로 보고 되었으며 이는 5년간 45만명이 늘어난 수치임. 특히 척추관 협착증의 경 우 5년 새 약 32.4% 늘어난 수치이다. 국민건강보험 2018년 수술 통계 연보에 따르면 내시경 하 척추 수술은 7,218건으로 전년도 5,108건에 비해 41.3% 증가하였으며, 그로 인한 진료비 역시 45.9% 증가하였음. 같은 기간 고식적 척추 수술 건수가 165,573건에서 169,706건으로 2.4% 증가한 것에 비하여 중대한 증가를 확인할 수 있어 척추 내시경 수술의 확장세가 두드러지고 있다고 할 수 있다. 또한 노인 인구의 증가에 따른 척추 질환의 유병률은 점차 증가될 것으로 보이며, 의료 비용의 부담도 같이 늘어날 것으로 예상된다.

요추 협착증에서의 후방 감압술 (Posterior decompression)과 요추 추간판 탈출증에서의 후방 접근 추간판 절제술 (discectomy)은 환자의 증상을 해결하기 위해 사용되고 있는 가장 고식적인 방법이다. 기존 고식적 방법은, 출혈이 많고, 수술 후 통증, 불안정성, 척추 주변 근육의 감소 등 문제가 있어 해부학적 구조물을 보존하기 위한 최소 침습 수술을 시행한다. 최소 침습수술의 대표적인 방법으로 편측 접근 후방 감압술 방법 (Unilateral laminectomy Bilateral decompression, ULBD)이 가장 많이 사용되고 있으며, 극상돌기 절개를 통한 방법, 내시경을 이용한 방법 등이 이용되고 있다. 최소 침습 수술은 기존 고식적 방법에 비해 많은 장점들이 있으며 임상적 결과도 기존 고식적 방법과 차이가 없다고 보고되어 있다.

최근 **내시경을 이용한 감압술과 추간판 절제술이 개발**되어 사용되고 있다. 내시경을 이용한 척추 수술에는 기구가 들어가는 개방공의 개수에 따라 단일공 내시경 수술법과 양방향 내시경 수술법으로 나뉜다. 1cm 이내의 피부 절개창을 통하여 수술부위에 접근하기 때문에 정상 구조물의 손상을 최소화 할 수 있어 수술 후 통증, 신경 유착 등의 합병증이 적다.

그러나 내시경을 이용한 척추 수술은 **후향적 연구로는 임상적 결과가 좋다고 보고된 바 있으나 다기관, 전향적, 무작위 배정 연구로는 아직 그 임상적 결과가 확인되지 않았다.** 또한 현재 요추 협착증 환자에서 시행하는 고식적 수술과 미세 현미경 수술은 보험 급여 항목으로 인정을 받아 수술이 필요한 환자들에게 적절하게 사용되고 있는 반면에, 척추 내시경 수술은 국내에서 아직 적절하게 인정받지 못하여 일부 추간판 탈출증 환자에서만 제한적으로 시행되고 있다. 국내에서 보고된 척추 내시경 수술 연구의 대부분은 후향적 연구이며 고식적 수술에 비한 내시경 **수술의 효용성과 안정성을 확립하기 위한 level 1 study는 부족하다.** 따라서 본 연구에서는 다기관 전향적 무작위배정 연구를 통해 내시경 수술의 효용성과 안정성을 임상적 결과가 확립된 고식적 후방 감압술의 임상결과와 비교해 보고자 한다.

9. 연구 유형

1) ■ 전향적 □ 후향적

2) 실험 연구 : ■ 무작위 대조군 연구 □ 비 무작위 연구

관찰 연구 : □ 코호트 연구 □ 사례대조 연구 □ 횡단적 연구 (□ 유병율 연구, □ 생태연구, □ 민감도와 특이도 연구)

10. 가설

척추관 협착증에서 내시경 수술 방법이 기존 고식적 수술과 비교하여 **임상적 결과의 동등성을 확인해 보고자 한다.**

11. 의료기기 또는 의약품 허가사항 외의 사용에 대한 임상시험으로서 식품의약품 안전처장의 임상시험 승인이 필요한 연구인가? [Investigational Device Exemption(IDE)]

□ 예 (식품의약품안전처장의 임상시험 승인이 필요한 연구) ■ 해당사항 없음

연구설계

12. 중재적 시술 혹은 기기 설명

1) 후궁간 경유 경피적 단일공 후방 감압술

아래 그림과 같이 내시경 기구와 척추 수술기구를 이용하여 후방 감압술을 시행한다. 정상 조직의 손상을 최소화 할 수

있다. 1cm 정도의 피부절개를 한 후 내시경을 삽입한 후 엑스레이상 위치를 확인 한 후 고주파를 이용하여 지혈을 하고 후궁을 노출한 후 내시경용 드릴을 이용하여 후관절의 손상을 최소화 하면서 후궁 절제를 시행한 후 황색인대가 노출되도록 한다. 황색인대 절제술을 시행하면서 추가적인 감압 및 시야 확보를 위해 필요한 경우 상부 척추의 후궁 뼈 아래 부분과 하부 척추의 후궁 뼈 윗부분을 조심스럽게 제거한다.

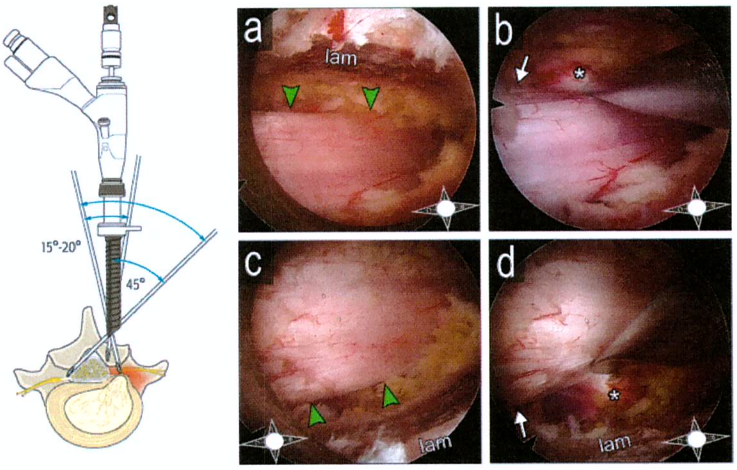

2) 고식적 후방 절제술

정중 절개선을 넣고 개방적으로 양측으로 접근하여 양측의 비후된 황색인대를 제거하는 방법으로서 현재 보편적인 후궁 절제술 방법이다. 수술 부위 소독 이후, 요추 측위 단순 방사선 촬영으로 수술 부위를 확인한다. 수술 부위의 정중선에서 장축으로 약 3 cm 길이로 피부 절개를 시행하며, 이 정도로 충분히 시야를 확보할 수 있다. 척추측방근육을 극상돌기, 후궁 및 척추관절로부터 박리 하여 근막피판을 젖힌 후 견인기를 이용하여 수술 시야를 확보한다.

추간공에서 황색인대 근위부는 상부 척추 횡돌기와 척추경(pedicle) 원위부 표면에서 확실하게 구분된다. 목표 구획의 황색인대 절제술을 시행할 때는 상부 척추 횡돌기의 원위부 말단 아래로 주행하는 신경근을 손상시키지 않도록 주의해야 한다. 황색인대 절제술을 시행하면서 추가적인 감압 및 시야 확보를 위해 상부 척추의 후궁 뼈 아래 부분과 하부 척추의 후궁 뼈 윗부분을 조심스럽게 제거하게 되는데, 이때 후관절이 손상받지 않도록 주의해야 한다. 이후 신경근이 확인하고, 신경근의 주행을 따라 말단부까지 충분히 감압을 하기 위해 하부 척추의 척추경 근위부까지 신경주행을 확인하며 감압을 시행한다. 반대쪽도 같은 방법으로 감압을 시행한 후 양측 신경근 주행이 자유로운지 확인한다. 이후 수술부위 지혈을 하고 수술부위 봉합 및 소독을 한 뒤 수술을 마친다.

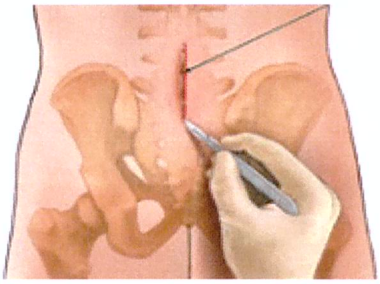
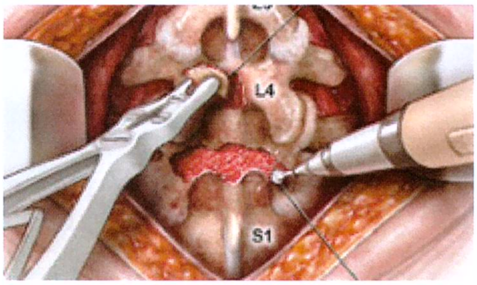

13. 적용 ■ 해당사항 없음

기기명	회사명	위치/장소	한국 식약처 승인	사용불가 시 대안
			□ 예 □ 아니오	
			□ 예 □ 아니오	

14. 연구 예상 기간

IRB 승인일부터~2024.12.31

**항목**	**예상시간**	**기간**
모집	개월	IRB 승인일부터~2024.12.31

임상 조사	개월	IRB 승인일부터~2024.12.31
자료 정리	2 개월	2024.01.01~2024.02.28
통계업무	2 개월	2024.03.01~2024.04.30
논문작성	8 개월	2024.05.01~2024.12.31
총	약 3 년 8 개월	IRB 승인일부터~2024.12.31

15. 환자 구성

자격요건		요추 중심관 협착증 Grade가 B이상으로 1-2 분절 후방 감압술을 시행하기로 한 사람<br>1 년 이상 추시 가능한 사람
전향연구 시	시술, 수술 등 치료 시행 예상 기간	IRB 승인일부터~2024.12.31
후향연구 시	시술, 수술 등의 치료 시행했던 기간	■ 해당사항 없음

표본크기와 산출배경

# 요추부 협착증 환자에서 단일공 내시경을 이용한 후방 감압술

- 목표 연구 대상자수: 120명 (시험군: 60명, 대조군: 60명, 탈락율 20 % 포함)
- Primary outcome: ODI (Oswestry disability index)
- 기존 논문[1]에 의하면 ODI의 MCID (Minimal clinical important difference)는 12.8 이었으며, 이전 연구[3]에서 decompressive laminectomy 수술 후 1년째 ODI 값의 standard deviation은 18.8이었다. 동등성 한계를 12.8로 정하고 alpha = 0.05, power = 0.90, two-sided 95% confidence interval, follow-up loss = 20%로 가정하면 각 군당 60명이 필요하며, 우리들병원에서는 40명의 연구대상자를 모집하고자 함.

Equivalence Tests for the Difference Between Two Means

Testing Equivalence of Two Means Using a Parallel-Group Design

Target Power	Actual Power	N1	N2	N	D	SD	Lower Equiv. Limit	Upper Equiv. Limit	Alpha
0.90	0.90438	48	48	96	0.0	18.8	-12.8	12.8	0.050

Summary Statements
An equivalence test of means using two one-sided tests on data from a parallel-group design with sample sizes of 48 in the reference group and 48 in the treatment group achieves 90% power at a 5.0% significance level when the true difference between the means is 0.0, the standard deviation is 18.8, and the equivalence limits are -12.8 and 12.8.

Dropout-Inflated Sample Size

	Sample Size			Dropout-Inflated Enrollment Sample Size			Expected Number of Dropouts		
Dropout Rate	N1	N2	N	N1'	N2'	N'	D1	D2	D
20%	48	48	96	60	60	120	12	12	24

Definitions
Dropout Rate (DR) is the percentage of subjects (or items) that are expected to be lost at random during the course of the study and for whom no response data will be collected (i.e. will be treated as "missing").
N1, N2, and N are the evaluable sample sizes at which power is computed. If N1 and N2 subjects are evaluated out of the N1' and N2' subjects that are enrolled in the study, the design will achieve the stated power.
N1', N2', and N' are the number of subjects that should be enrolled in the study in order to end up with N1, N2, and N evaluable subjects, based on the assumed dropout rate. After solving for N1 and N2, N1' and N2' are calculated by inflating N1 and N2 using the formulas N1' = N1 / (1 - DR) and N2' = N2 / (1 - DR), with N1' and N2' always rounded up. (See Julious, S.A. (2010) pages 52-53, or Chow, S.C., Shao, J., and Wang, H. (2008) pages 39-40.)
D1, D2, and D are the expected number of dropouts. D1 = N1' - N1, D2 = N2' - N2, and D = D1 + D2.

Procedure Input Settings

Design Tab

Solve For:	Sample Size
Power:	0.90
Alpha:	0.05
Group Allocation:	Equal (N1 = N2)
|EU| (Upper Equivalence Limit):	12.8
-|EL| (Lower Equivalence Limit):	-Upper Limit
D (True Difference):	0
SD (Standard Deviation):	18.8

포함기준	A. 20세부터 80세<br>B. 요추 중심관 협착증 Grade가 B 이상으로 1-2 분절 후방 감압술을 시행하기로 한 사람<br>C. 1년 이상 추시 가능한 사람<br>D. 본인(서명이 가능한 경우에 한함) 또는 법적 대리인이 임상시험의 내용을 충분히 이해한 후 동의서에 서명한 연구 대상자
제외기준	A. Meyer Gr II 이상의 척추전방전위증<br>B. 이전에 같은 분절 수술 시행한 사람<br>C. 요추 퇴행성 측만증 (Cobb angle > 20도)<br>D. 협착증의 원인이 퇴행성이 아니거나 추간판탈출증에 의한 경우<br>E. 요추 협착증 이환부위에 다른 척추 질환이 있는 경우 (강직성 척추염, 종양, 압박 골절 등)<br>F. 정신과적 질환 (치매, 정신지체자, 심한 약물 중독 등)<br>G. 연구에 참여하기 거부한 사람<br>H. 기타 임상시험 담당자가 임상시험 대상으로 부적당하다고 판단하는 환자
대조군	**고식적 후방 절제술**
모집방법	외래내원환자
환자 배정(임의추출 등)	- 다기관, 평가자 눈가림, 무작위배정, 대조군 비교, 전향적 임상시험으로 전향적, 무작위 배정 연구를 시행한다.<br>- 각 세부 과제에 배정된 환자 군에 따라 내시경군 (1군)과 고식적 수술군 (2군)으로 나누어 연구를 시행하고자 한다.<br>- 수술 방법의 차이 외에 두 군은 수술 전 후 동일한 처치와 추시관찰 (수술일, 수술 후 2주, 3개월, 6개월, 1년)을 하면서 방사선학적, 임상적 수술 결과에 대한 변수를 기록하여 두 군간의 차이를 비교한다. 이 연구에 등록 예정인 환자는 연구에 참여하기 전에 연구 담당자로부터 연구에 대한 목적, 방법을 듣고 동의서에 서명함으로써 연구에 등록이 된다. 스크리닝 배정 번호는 일련의 여섯 자리 번호로 부여되며 무작위 배정번호의 첫째 자리는 임상시험실시기관을 의미하고, 둘째 자리는 세부과제를 의미하며, 세번째 자리는 Screening의 S, 나머지 세 자리는 등록의 일련번호를 의미한다. (예시: B1S-001 -> 분당서울대병원의 1세부 수술의 첫 스크리닝 등록 환자)<br>- 연구에 자의적으로 참여할 것을 동의하고 동의서에 서명한 연구대상자 중 포함 및 제외 기준을 만족하는 연구대상자는 두 군 중 한 군에 1:1의 비율로 각각 무작위로 배정된다.<br>- 무작위 배정은 permuted block randomization 방법을 이용한다. 무작위 배정은 Web-based eCRF인 iCReaT을 이용하여 연구대상자 번호 1번부터 순차적으로 적용한다. 연구자는 연구대상자의 무작위 배정 결과에 따라 해당 수술을 시행할 예정이다. Bias를 최소화하기 위해 randomization은 연구원이 시행을 하며, 수술 직전 무작위배정 코드를 수술자에게 통보한다.<br>이 연구는 이중 맹검법을 적용하기에는 한계가 있다. 연구자 스스로 어떠한 술식을 사용했는지 알게 되며, 환자는 수술 상처를 확인해서 수술 방법을 알게 된다. 따라서 평가자에게만 눈가림 방법을 적용하는 단일 맹검법을 적용하며, 임상 관찰자는

	수술을 시행하지 않은 제3자로 한다.<br>- 무작위 배정 번호는 일련의 다섯자리 번호로 부여되며 무작위 배정번호의 첫째 자리는 임상시험실시기관을 의미하고, 둘째 자리는 세부과제를 의미하며, 나머지 세 자리는 등록의 일련번호를 의미한다. (예시: B1-001 -> 분당서울대병원의 1세부 수술의 첫 등록 환자)
조사 방법	- 모든 기본적인 검진이나 설문을 수술 전과 수술, 수술 후 2주, 3개월, 6개월 및 1년 추시시 평가한다. 수술 전 이 연구대상자들을 대상으로 수술 전 방사선학적 평가, 임상적 신체검진 (Age, Gender) 및 병력청취 (과거력, 수술력)를 시행하고, 수술 전 검사상 Lab (CBC, Routine chemistry)을 확인한다. 수술 전과 수술 직후 (X-ray, MRI 혹은 CT), 수술 후 2주, 3개월, 6개월, 12개월 영상의학적 검사 (X-ray)를 통해 합병증 유무를 확인한다. 수술 전, 수술 후 2주, 3개월, 6개월, 12개월째 VAS, ODI, EQ5D, 보행, 만족도, POSAS를 설문조사 하여 임상적 평가를 시행한다. 수술 직후 기타 수술과 관련된 항목 (수술 후 출혈량, 수술 시간, 입원 기간, 수술 후 PCA 사용 정도, 수술 후 1일 째 Creatine Kinase (CK, CPK))을 측정하여 수술과 관련된 항목을 비교한다.<br>- 단, 불가항력적인 이유 (예를 들면 Covid 19로 인한 안전상의 이슈 등)로 예정된 시기에 방문하지못할 경우 현행법이 허락하는 비대면 진료 혹은 전화 설문으로 대신할 수 있다.

16. 결과 측정

임상적	VAS, ODI, EQ5D<br>이학적 및 신경학적 검사 결과<br>혈액 검사
방사선학적	요추 및 전척추 일반 방사선 촬영<br>요추 전산화 단층 촬영<br>요추 자기공명영상
기타	수술만족도, POSAS 흉터 설문

17. 자료 관리

통계 분석 방법	**VAS, EQ5D-5L, ODI, 보행가능 시간**<br>수술 전 대비 수술 후 기간별 임상적 평가 점수의 변화양상<br><br>통계적 분석 방법 : 각 군 별로 수술 전 대비 수술 후, 수술 전후 차이와 양 군 간 변화양상의 차이를 repeated measures ANOVA로 분석한다. 또한 수술 후 각 시점에서 집단내 또는 집단간 차 이의 구체적인 비교는 t-test로 비교하되, 검정의 다중성을 고려하여 조정된 유의수준을 적용한다.<br><br>**수술 후 만족도, 수술 후 일상생활복귀 기간, 수술 후 흉터 (POSAS scale)**<br><br>통계적 분석 방법: 수술 후 최종 추적 관찰 당시의 수술 후 만족 정도와 수술 후 일상생활복귀기간, POSAS scale score를 t-test를 이용하여 양 군 간 비교 평가한다.<br><br>**영상 검사**<br>단순 방사선 검사상 최종 추시시 합병증 유무 (전방전위증 발생, 퇴행성 변화의 진행 등), 수술 후MRI (혹은 CT)를 통한 수술 결과 분석<br><br>통계적 분석 방법: 각 군 별로 합병증 발생 유무를 확인, 수술 후 MRI 혹은 CT를 통한 추간판 절제 정도를 Chi-square test 혹은 t-test를 이용하여 양 군 간 차이의 정도를 평가한다.<br><br>**수술과 관련된 기타 항목**<br>수술과 관련된 기타 항목에 대한 양군간 비교. [수술 incision크기, 수술 후 출혈량, 수술 시간, 입원 기간 (시간), 수술 후 (수술 후 ~퇴원시) PCA 사용 정도 (Fentanyl use, mcg), 수술 후 1일째 Creatine Kinase (CK, CPK)]<br><br>통계적 분석 방법: 양 군간 차이를 Chi-square test 혹은 t-test를 이용하여 양 군 간 차이의 정도를 평가한다<br><br>**결과분석의 일반적 원칙**<br>· 연속형 변수의 경우에는 기술 통계량(시험대상자 수, 평균, 표준편차, 중앙값, 최소값, 최대값)을 제시하고, 범주형 변수에 대해서는 빈도(N) 및 비율(%)을 제시

분석군의 처리

·Safety set, FAS (full analysis set)와 PP (per protocol)로 나눔

·유효성에 대한 자료는 FAS 및 PP를 모두 사용하여 분석하며, 유효성 평가변수에 대한 최종판정은 modified ITT(intention to treat)인 FAS 분석으로 실시

·안전성에 대한 자료는 Safety set을 대상으로 분석

·유효성 평가 자료 중 결측치가 발생한 경우에는 수치를 대체하지 않고 결측 처리

분석군의 정의

·FAS (Full Analysis Set) 대상군: 선정/제외 기준에 적합하였고, 무작위 배정되어 시험기간 동안 한 번이라도 수술받은 시험 대상자를 대상으로 한다. 유효성 분석 시에는 실제 수술과 상관없이 무작위 배정받은 치료군으로 포함하여 분석한다.

·PP (Per Protocol Set) 대상군: FAS 분석군 중에서 중대한 시험계획서 위반 없이 시험을 완료한 시험 대상자를 대상으로 한다. 단 중지기준에 따라 임상시험이 중지된 경우는 임상시험을 완료한 것으로 보고 PP분석에 포함한다. 중대한 임상시험계획서 위반에 대한 항목은 13-5절을 참고한다.

·안전성 (Safety Set) 대상군: 무작위 배정되어 시험기간 동안 한 번이라도 수술받은 시험 대상자를 대상으로 한다. 안전성 분석 시에는 실제로 수술받은 치료군으로 포함하여 분석한다.

증례기록지 형식	증례 기록지(환자 정보 수집 양식): 첨부
1) 결측치	·· 일차 유효성 분석 시, 중도 탈락등의 사유로 결측치가 발생하는 경우 해당 데이터는 실패로 간주하여 처리하는 NRI (non-responder impuation) 방법을 사용<br>· 중지기준에 따라 임상시험이 중지된 경우는 결측치 처리하고<br>· 이차 유효성 분석 시 결측치가 발생 했을시에는 LOCF 방법을 사용<br>· Safety set 분석은 결측치 보정 없이 관측치 그대로(OC method) 사용

18. 예상

본 연구에서는 내시경 수술의 효용성과 안정성이 임상적 결과가 확립된 고식적 후방 감압술의 임상결과와 비교해 차이가 없음을 확인함으로서 귀중한 자료가 될 것이다.

윤리적 문제

19. 사전 동의 (환자와 의사 모두에게)

■ 필요 ☐ 불필요

20. 예상 비용(연구에 소요될 예상비용)

항목	상세사항	수량	예상 총액
유인물비	N/A	N/A	N/A
정보 DB 사용료	N/A	N/A	N/A
재료비(연구자재 등)	N/A	N/A	N/A
전산처리비	N/A	N/A	N/A
수용비(전화/전기 등)	N/A	N/A	N/A
IRB 심사비	초기 심의비	500,000	500,000
간접경비	N/A	N/A	N/A
합계	N/A	N/A	500,000

21. 참여에 대한 환자의 이익

- N/A

22. 이해상충 공개 진술

■ 본인은 이 논문에서 언급한 주제, 자료 혹은 단체와 어떠한 재정적 관계 혹은 이해상충관계가 없음을 증명합니다.

☐ 본인은 하나 혹은 그 이상의 이해상충관계가 있음을 선언합니다.

이해상충관계 공개:
(*예: 고용, 자문 회사 소속, 주식자본 소유, 수수료*)
( )

23. 대체 치료법

- ■ 해당사항 없음

24. 잠재적 위험성(필요 할 경우, 비율 기재)

- ■ 해당사항 없음

25. 잠재적 이득(필요 할 경우, 비율 기재)

- ■ 해당사항 없음

26. 부작용 보고& 사용상의 주의사항

*** 예측되는 부작용/위험 및 대처 방안**

본 연구에서 시행할 요추 후방 감압술은 요추부 척추관 협착증 치료로서 일반적으로 시행하는 표준 치료법으로, 일반적인 척추 수술에 대한 합병증(출혈, 감염, 신경손상 등) 이외에 본 연구로 인해서 추가되는 합병증은 없다.

하지만, 만약 본 연구와 관련된 부작용 발생시 연구 담당자는 다음과 같은 조치를 취한다.

① 의사는 부작용을 발견하였을 경우, 즉시 IRB에 신고한다.

② 의사는 필요한 의학적 조치를 취한다.

본 연구로 인해서 연구 대상자에게 피해가 발생하여 응급조치가 필요한 경우, 가능한 빨리 응급조치를 시행한다. 또한 중대한 유해사례 발생 시 빠르고 적절한 조치를 취하여 가능한 피해를 최소화할 것이다.

단. 일반적인 요추 수술 후 예상되는 합병증의 경우는 "본 연구로 인한" 합병증으로 간주하지 않는다.

27. 안전성 평가 규범& 평가 방법 & 부작용을 포함한 보고 방법

임상연구기간 동안 발생된 이상반응은 증상이 해결될 때까지 또는 안정될 때까지 추적 관찰한다. 발생되는 이상반응은 MED-DRA(Medical Dictionary for regulatory activities)을 이용한 선호용어로 코드화한다. 치료 후 발생한 모든 이상반응(AEs), 중대한 이상반응(SAEs), 임상연구 치료와 관련된 예상하지 못한 이상반응(UAEs)을 중증도에 따라 시 작 시점별로 선호용어를 이용하여 요약하고 도표로 작성하며, 시험대상자의 자발적인 보고 및 내원 시 확인하여 기록한다. 기록시 명칭과 지속기간, 증상의 범위, 정도, 의료기기와의 인과관계, 추가적인 치료, 이상반응의 결과, 중대성 등에 대하여 증례기록서의 Special Form에 구체적으로 기술한다.

통계적 분석 방법: 본 연구에서 이상반응은 이식 수술 전에는 관찰되지 않았던 증상 또는 징후가 수술 후 새로 나타나거나 악화된 경우로서, 기기와의 관련성에 무관하게 나타난 모든 증후(sign), 증상(symptom), 질병을 포함한다. 이상반응이 나타나면 관련 증상명, 발현시기, 지속기간, 증상의 정도 및 시험기기와의 인과관계 등에 대하여 증례기록서에 기록하고 발생한 이상반응에 대해서는 각 이상반응 별로 건수와 해당 연구 대상자 수를 계산하며, 이상반응 발현율과 95% 양측 신뢰구간을 제시한다. 양 군간 이상반응률의 차이는 카이제곱검정 또는 Fisher의 exact test로 비교한다.

28. 암 관련 연구

- ■ 해당사항 없음

29. 피해자 보상규정

- '연구대상자 피해 보상에 대한 규약'(첨부), 보험 증권(첨부)

30. 환자의 의료기록과 정보의 비밀보장

모든 연구대상자 정보에 대하여 비밀이 보장되어야 한다. 연구와 관련된 모든 기록물은 환자의 의무기록번호 및 병록 번호는 주관연구자의 책임하에 별도의 파일로 보관하며 이를 코드화하여 연구데이터를 통하여 개인 신상 확인이 불가능하도록 관리한다. 또는 연구데이터는 패스워드가 걸린 파일에 저장하여 잠금 장치가 있는 연구실에 보관하도록 한다. 각 시험대상자의 개인 신상정보가 확인되지 않도록 식별코드 번호로 번호를 부여하며 성명은 이니셜로 기록한다. 연구책임자는 본 연구와 관련하여 모든 정보에 대해 비밀보장을 해야만 한다. 생명윤리법 시행규칙 제 15조에 따라 연구 관련 기록을 연구가 종료된 시점부터 3년간 보관하여야 하며, 보관기간이 지나면 개인정보보호법 시행령 제 16조에 따라 파기하도록 한다. 다만, 후속 연구, 기록, 축적 등을 위해 보관 계획이 변경된다면 추가 보관하도록 한다. 본 연구의 정보는 오직 임상시험심사위원회나 관련된 연구진에게만 개방할 수 있다. 시험책임자는 본 시험의 결과를 등록, 발표 및 의약학 전문가들을 위한 정보제공 등의 목적으로만 사용할 수 있다.

- 본인은 이로써 환자정보의 비밀보장을 약속합니다. 서명 ____________________

행정 업무

31. 협조 부서

- 학술홍보팀

32. 자료와 도구의 위치

- N/A

33. 환자에게 결과 통지

- ■ 해당사항 없음

연구책임자 서명 날짜

□ 본 연구는 IRB 승인이 필요하지 않습니다.

기관심의윤리위원회의 승인 □ 승인 (날짜:) □ 거절

식품의약품안전처의 의료기기 또는 의약품 품목 허가사항 이외의 사용은 식품의약품안전처에 계획서 제출이 필요합니다.

□ 필요 없음, 의료기기 또는 의약품의 허가사항 범위내의 임상시험

□ 의료기기 또는 의약품 허가사항 외의 사용에 대한 임상시험으로 식품의약품 안전처장의 임상시험 승인 획득

□ 의료기기 또는 의약품 허가사항 외의 사용에 대한 임상시험이나 식품의약품 안전처장의 임상시험 승인 미획득

병원장 서명 (병원장) Date

이사장 서명 (이사장) Date

APPENDIX

1. Reference (관련 논문의 초록): 첨부
2. Case report form (증례 기록지: 환자 데이터 정리 양식): 첨부
3. Informed Consent (연구 협조 동의서: 환자, 타 주치의)
4. PI(연구책임자) 이력서

1. Reference (관련 논문의 초록)

1. Wing P, Cheung H, King C, Wong H, Lau ST, Cost analysis comparison between conventional microsurgical decompression and full-endoscopic interlaminar decompression for lumbar spinal stenosis surgery. J Spine Surg. 2020 Dec;6(4):721-728. doi: 10.21037/jss-20-552.
2. Chen KT, Choi KC, Song MS, Jabri H, Lokanath YK, Kim JS. Hybrid Interlaminar Endoscopic Lumbar Decompression in Disc Herniation Combined With Spinal Stenosis. Oper Neurosurg (Hagerstown). 2021 Feb 16;20(3):E168-E174. doi: 10.1093/ons/opaa360.
