# Supplementary material for: Evaluation of the efficacy and safety of conventional and interlaminar full-endoscopic decompressive laminectomy to treat lumbar spinal stenosis (ENDO-F trial): Protocol for a prospective, randomized, multicenter trial
Source: PLoS One. 2023 Apr 5;18(4):e0283924. doi: 10.1371/journal.pone.0283924 (PMC10075401; doi:10.1371/journal.pone.0283924)
Supplement: Attachment — Submitted filename: Response to Reviewers.docx [file pone.0283924.s008.docx]

**Response to Reviewers**

**Comments from Reviewer #1**

Comment 1: there is no mention about the surgeons’ experience with the endoscopic spinal technique. Please consider this aspect.

Response: We would like to thank Reviewer 1 for the time and efforts in reviewing our manuscript and for providing comments, which have considerably helped us improve our manuscript. We have made revisions based on the comments and have provided our point-by-point responses below. The revisions are indicated using track changes in the revised manuscript. We hope that our responses and revisions appropriately address the reviewer’s comments.

We agree with the suggestion to mention the surgeon’s experience and have included the following information in the revised manuscript.

“Faculty members with clinical experience of 7–12 years in both endoscopic and conventional surgeries will participate.” (Lines 208–209 of the revised manuscript)

Specifically, these experts perform 5–10 endoscopic surgeries per month.

“As described above, surgeons’ experience and technique of endoscopic spinal surgery may present little differences. Although there may be differences in surgical outcomes across the surgeons enrolled in this multicenter study, the differences will be verified using the Cochran–Mantel–Haenszel test.”

We have provided this information in the revised manuscript (Lines 279–282).

Comment 2: it is necessary to define the outcomes in regard to the age, underlining the “statistical difference” between the two groups.

Response: We agree with the reviewer’s comment. We have provided the following information in the revised manuscript (Lines 282–285).

“Differences between the two groups of variables used will be verified at baseline, and additional necessary analysis will be performed using analysis of covariance (ANCOVA) or mixed model analysis. The difference in age between the two groups will be verified by t-test, and ANCOVA will be performed using age as a covariate in ODI score analysis.”

Comment 3:  it is interesting to define the surgical time difference between the two groups, in order to suggest the endoscopic technique for the treatment of the elderly patients.

Response: We agree with the reviewer’s comment. We have provided the following information in the revised manuscript (Lines 286–288).

“The difference in surgical time between the two groups will be verified by t-test. If necessary, ANCOVA will be performed using age as a covariate in surgical time analysis to suggest the endoscopic technique for the treatment of elderly patients.”
